# Low-carbohydrate diet score and chronic obstructive pulmonary disease: a machine learning analysis of NHANES data

**DOI:** 10.3389/fnut.2024.1519782

**Published:** 2024-12-18

**Authors:** Xin Zhang, Jipeng Mo, Kaiyu Yang, Tiewu Tan, Cuiping Zhao, Hui Qin

**Affiliations:** ^1^Department of Emergency Medicine, The Affiliated Changzhou No.2 People’s Hospital of Nanjing Medical University, The Third Affiliated Hospital of Nanjing Medical University, Nanjing Medical University, Changzhou, China; ^2^Department of Intensive Care Medicine, The Affiliated Changzhou No.2 People’s Hospital of Nanjing Medical University, The Third Affiliated Hospital of Nanjing Medical University, Nanjing Medical University, Changzhou, China; ^3^Department of Geriatrics, The Affiliated Changzhou No.2 People’s Hospital of Nanjing Medical University, The Third Affiliated Hospital of Nanjing Medical University, Nanjing Medical University, Changzhou, China

**Keywords:** NHANES, low-carbohydrate diet score, chronic obstructive pulmonary disease, cross-sectional study, machine learning

## Abstract

**Background:**

Recent research has identified the Low-Carbohydrate Diet (LCD) score as a novel biomarker, with studies showing that LCDs can reduce carbon dioxide retention, potentially improving lung function. While the link between the LCD score and chronic obstructive pulmonary disease (COPD) has been explored, its relevance in the US population remains uncertain. This study aims to explore the association between the LCD score and the likelihood of COPD prevalence in this population.

**Methods:**

Data from 16,030 participants in the National Health and Nutrition Examination Survey (NHANES) collected between 2007 and 2023 were analyzed to examine the relationship between LCD score and COPD. Propensity score matching (PSM) was employed to reduce baseline bias. Weighted multivariable logistic regression models were applied, and restricted cubic spline (RCS) regression was used to explore possible nonlinear relationships. Subgroup analyses were performed to evaluate the robustness of the results. Additionally, we employed eight machine learning methods—Boost Tree, Decision Tree, Logistic Regression, MLP, Naive Bayes, KNN, Random Forest, and SVM RBF—to build predictive models and evaluate their performance. Based on the best-performing model, we further examined variable importance and model accuracy.

**Results:**

Upon controlling for variables, the LCD score demonstrated a strong correlation with the odds of COPD prevalence. In compared to the lowest quartile, the adjusted odds ratios (ORs) for the high quartile were 0.77 (95% CI: 0.63, 0.95), 0.74 (95% CI: 0.59, 0.93), and 0.61 (95% CI: 0.48, 0.78). RCS analysis demonstrated a linear inverse relationship between the LCD score and the odds of COPD prevalence. Furthermore, the random forest model exhibited robust predictive efficacy, with an area under the curve (AUC) of 71.6%.

**Conclusion:**

Our study of American adults indicates that adherence to the LCD may be linked to lower odds of COPD prevalence. These findings underscore the important role of the LCD score as a tool for enhancing COPD prevention efforts within the general population. Nonetheless, additional prospective cohort studies are required to assess and validate these results.

## 1 Introduction

Chronic obstructive pulmonary disease (COPD) is a common long-term respiratory condition characterized by persistent airflow limitation, typically resulting from ongoing inflammation of the airways and lung tissue (1, 2). COPD has emerged as a significant factor in global morbidity and mortality rates, exerting considerable impacts on public health and economic systems (3). Based on the global burden of disease research, there are currently over 200 million COPD patients worldwide, a figure projected to continue rising (4). While smoking and long-term exposure to harmful gases are recognized as the primary risk factors (5), a notable proportion of COPD patients are non-smokers (6). Research indicates that approximately half of all COPD cases are associated with non-tobacco factors (7). This observation has prompted researchers to investigate other potential contributors to the onset and evolution of the disease, particularly the role of dietary imbalance (8–10).

Low-carbohydrate diets (LCD), which reduce carbohydrate intake while moderately increasing the proportion of proteins and fats, have gained widespread attention in recent years (11). The differences in respiratory quotient (RQ) for various nutrients indicate that long-term inappropriate nutritional intake may adversely affect lung health (12, 13). Carbohydrates, as a major energy source for the body (14), produce higher respiratory quotients and carbon dioxide (CO2) during metabolism, thereby increasing the burden on the respiratory system (15). Studies have shown that excessive carbohydrate intake is closely related to respiratory health, particularly in individuals with underlying conditions or those at high risk. Reducing carbohydrate intake can effectively reduce CO2 production, thereby alleviating respiratory stress (16, 17). Moreover, low-carbohydrate, high-fat diets are considered beneficial for alleviating CO2 retention in the lungs of COPD patients, improving nutritional status, enhancing exercise capacity, and increasing lung function (18, 19). Therefore, an evaluation system for low-carbohydrate diets, by integrating these nutritional components, provides a novel perspective and helps to deepen our understanding of the potential impact of nutritional regulation on the odds of COPD prevalence.

Chronic illnesses, such as diabetes, metabolic syndrome, coronary artery disease, and cognitive decline, are significantly correlated with the LCD score (20–23). Although previous studies have indicated that low-carbohydrate diets may influence the odds of developing COPD (24), research exploring the relationship between the LCD score and COPD remains insufficient. Current research is constrained by restricted sample sizes and a focus on specific geographic regions; furthermore, the potential non-linear relationship between the LCD score and the likelihood of COPD prevalence has not yet been examined. In light of these limitations, research utilizes data from the National Health and Nutrition Examination Survey (NHANES) spanning 2007 to 2023 to perform a cross-sectional analysis investigating the potential association between the LCD score and the odds of COPD prevalence.

## 2 Methods

### 2.1 Study cohort and data collection

The NHANES, administered biennially by the US Centers for Disease Control and Prevention (CDC), evaluates the health and nutritional status of the US population. Utilizing a multi-stage probability sampling method, NHANES chooses roughly 5,000 participants each year from varied places across the country, guaranteeing representativeness (25, 26). All subjects granted informed consent before their enrollment in the study. The survey collects extensive data, including demographic information, questionnaire responses, medical examinations, laboratory results, and dietary intake data, to uphold data integrity and ethical standards. Comprehensive information regarding the survey’s design and analytical methodology is available on the CDC website.

**Figure 1:**
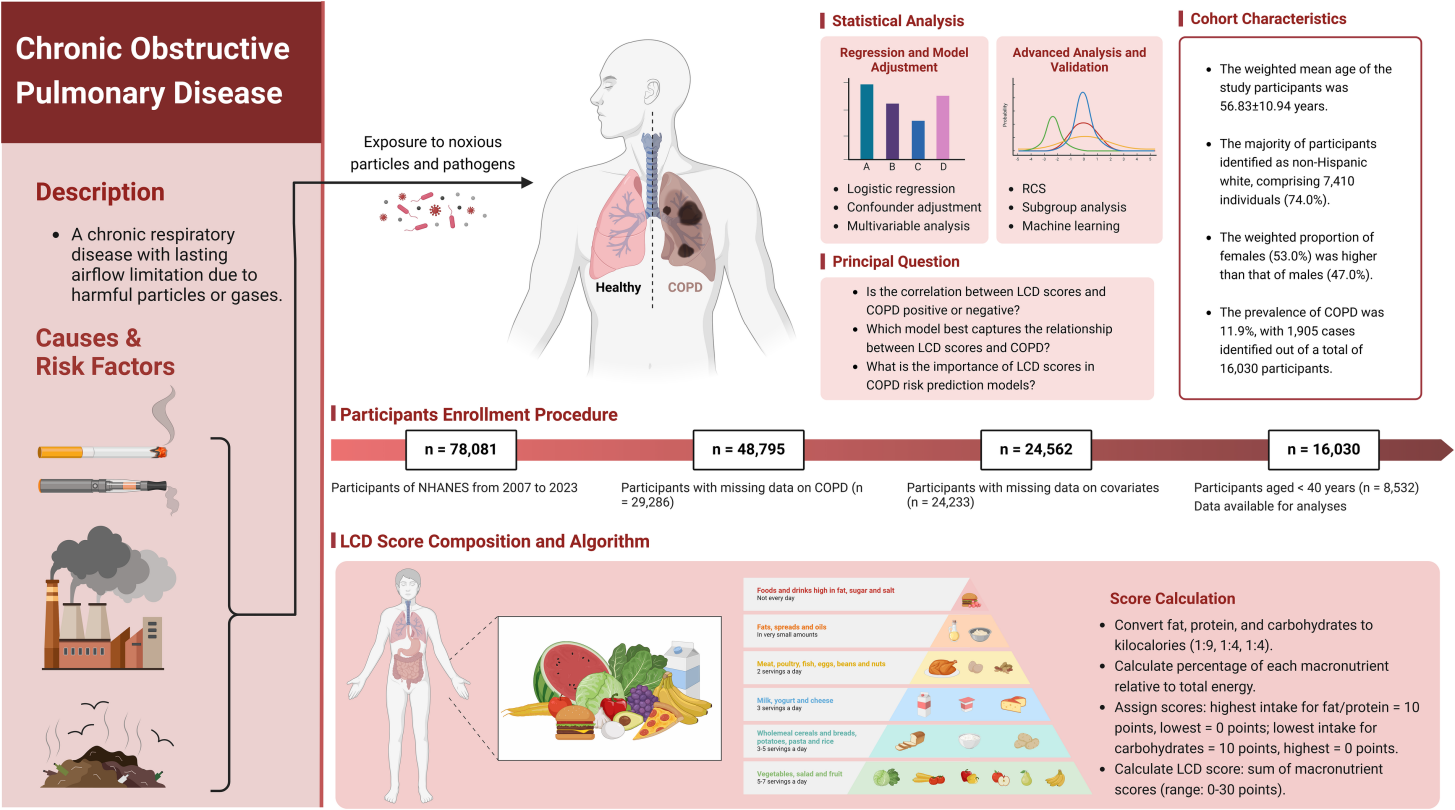
Scheme of the study’s objectives and the participant selection process. Our objective is to assess the relationship between LCD score and adults with COPD. LCD, Low-Carbohydrate Diet; COPD, Chronic Obstructive Pulmonary Disease; NHANES, National Health and Nutrition Examination Survey. Created with BioRender.com.

The current analysis utilized cross-sectional data from 78,081 participants across 7.6 consecutive NHANES cycles (2007–2023). We applied specific exclusion criteria: (1) participants without COPD diagnosis data (*n* = 29,286); (2) individuals with missing covariate information, including education level, marital status, poverty-to-income ratio (PIR), body mass index (BMI), waist circumference, standing height, physical activity, smoking status, hypertension, congestive heart failure, coronary heart disease, heart attack, stroke, magnesium intake, calcium intake, vitamin D intake, and intake of fat, protein, carbohydrates, and energy (*n* = 24,233); and (3) those younger than 40 years (*n* = 8,532). After implementing these criteria, 16,030 participants remained eligible for further analysis. Figure 1 presents a comprehensive flowchart of the participant recruitment procedure.

### 2.2 Collection of data

This study identified several confounding variables based on existing research and clinical evaluations, including age, sex, race/ethnicity, education level, marital status, poverty-to-income ratio (PIR), body mass index (BMI), waist circumference, waist-to-height ratio (WHtR), physical activity levels, smoking habits, hypertension, diabetes mellitus (DM), cardiovascular disease (CVD), and average dietary intake of magnesium, calcium, and vitamin D. The following groups were used to categorize self-reported race/ethnicity: Mexican American, other Hispanic, non-Hispanic White, non-Hispanic Black, and other race. There were two categories for marital status: married and unmarried. Educational attainment was categorized into three levels: less than high school, high school graduate, and higher than high school. Economic status was assessed using the PIR, and BMI was computed from weight measured against height. Waist circumference, height, and weight were measured following the guidelines outlined in the *Anthropometry Procedures Manual*, which incorporates rigorous quality assurance (QA) and quality control (QC) procedures to minimize measurement errors. Smoking status was categorized into non-smokers (individuals who had smoked fewer than 100 cigarettes in their lifetime) and smokers (individuals who had smoked more than 100 cigarettes and were currently smoking). Physical activity was assessed using the first question of the *Global physical activity questionnaire* (GPAQ), which asks: “Does your work involve vigorous-intensity activity that causes large increases in breathing or heart rate, such as carrying or lifting heavy loads, digging, or construction work, for at least 10 min continuously?” Individuals participating in a minimum of 10 min of such exercise were designated as active, whereas those engaging in less were classed as inactive. The history of CVD was derived from self-reported diagnoses of congestive heart failure, coronary heart disease, heart disease, or stroke. Hypertension and diabetes were self-reported conditions, with diabetes defined by any of the following criteria: an HbA1c level surpassing 6.5%, a diagnosis from a healthcare professional, fasting glucose levels of 7.0 mmol/L or above, random or 2-h oral glucose tolerance test (OGTT) glucose levels of 11.1 mmol/L or more, or the administration of diabetes medications or insulin. For comprehensive details regarding these variables, please refer to the NHANES website.

### 2.3 Dietary intake evaluation

Two 24-h dietary recalls’ average results guided the evaluation of food intake. The first interview took place at a mobile examination center (MEC), then 3 to 10 days later the second one over the phone. The Food and Nutrition Database for Dietary Studies (FNDDS) was employed to calculate the daily total energy and nutrient intake based on the consumption of foods and beverages reported within the 24 h preceding each interview (27, 28).

### 2.4 Low-carbohydrate diet score

By calculating the average total energy and nutrient intake from both interviews, we categorized participants’ carbohydrate, protein, and fat energy percentages into 11 tiers (Supplementary Table 1). The LCD score was derived from a comprehensive assessment of these three macronutrients. Initially, the consumption of each gram of fat, protein, and carbs was converted to kilocalories with the corresponding conversion factors of 1:9 for fat and 1:4 for both protein and carbohydrates. Subsequently, we calculated the proportion of each macronutrient in relation to total energy consumption. The lowest intake % for carbohydrates scored 10, while the maximum scored 0; in contrast, the highest intake percentage for fat and protein scored 10, and the lowest scored 0 (11). Ultimately, the LCD score was the aggregate of the values for the three macronutrients, ranging from 0 to 30, where elevated scores signify reduced carbohydrate consumption and increased fat and protein intake. In this study, LCD scores were divided into four groups using the 25th, 50th, and 75th percentiles.

### 2.5 Chronic obstructive pulmonary disease

To thoroughly assess our target population, we utilized two distinct diagnostic criteria based on the NHANES database (29, 30). First, we assessed medical history by asking participants, “Has a doctor or other health professional ever told you or the sample person (SP) that you/he/she had COPD?” Individuals who answered “yes” were categorized as having COPD, while those who responded “no” were categorized as not having the condition. Second, we performed pulmonary function tests, necessitating participants to have an FEV1/FVC ratio below 70% after inhaling a bronchodilator. Participants fulfilling this condition were classified as having COPD, whereas those who did not were classified as not having the disease. The reliability of these diagnostic criteria has been validated in previous studies, confirming the robustness of our inclusion standards.

### 2.6 Statistical analysis

Propensity score matching (PSM) was conducted utilizing a 1:1 nearest-neighbor approach to reduce bias and account for potential confounding baseline variables between the COPD and non-COPD cohorts. Matching variables included age, sex, race, education level, PIR, BMI, WHtR, smoking status, hypertension, diabetes, congestive heart failure, coronary heart disease, heart disease, stroke, and magnesium intake. After matching, if the *p*-values for intergroup differences exceeded 0.05, it suggested no statistically significant baseline differences, indicating that the matched groups achieved reasonable balance in baseline characteristics (31–33). In accordance with the NHANES analytic standards (accessed on March 4, 2024), all analyses included sample weights, clustering, and stratification to assure national representativeness of the US civilian non-institutionalized population with COPD and to get precise variance estimation (34, 35). For data with a normal distribution, continuous variables are expressed as mean ± standard deviation (Mean ± SD), whereas for data that do not follow a normal distribution, they are presented as median (IQR). Categorical variables are given as counts and percentages [n (%)]. Comparisons across groups were conducted utilizing weighted Student’s t-tests, Mann–Whitney U tests, and Chi-square tests, contingent upon the variable type and distribution.

Multivariable logistic regression models were utilized to evaluate the relationship between the LCD score and the likelihood of COPD prevalence, comprising one unadjusted (crude) model and two more adjusted models (Model I and Model II). Model I was adjusted for demographic variables such as age, sex, race/ethnicity, education level, marital status, and PIR. Model II was further adjusted for additional potential confounders. To explore the possible non-linear association between the LCD score and COPD, we utilized restricted cubic spline (RCS) regression with knots placed at the 5th, 35th, 65th, and 95th percentiles of the LCD score distribution. Additionally, subgroup analyses were conducted to examine the correlation between the LCD score and the odds of COPD prevalence across different strata, including age, sex, race/ethnicity, marital status, education level, PIR, smoking status, diabetes, hypertension, congestive heart failure, coronary heart disease, heart disease, stroke, physical activity, and BMI. Ultimately, we analyzed the interplay between the LCD score and the stratification variables by logistic regression to investigate the correlation between the LCD score and the odds of COPD prevalence within each subgroup.

Eight machine learning algorithms—Boost Tree, Decision Tree, Logistic Regression, Multilayer Perceptron (MLP), Naive Bayes, K-Nearest Neighbors, Random Forest, and Support Vector Machine with a Radial Basis Function (SVM RBF)—were utilized to generate receiver operating characteristic (ROC) curves, calibration plots, and decision curve analyses (DCA) (36, 37). These tools were used to assess model sensitivity, specificity, predictive accuracy, and decision-making value (38). To guarantee a rigorous performance assessment, the data was randomly divided between training and testing sets, utilizing five-fold cross-validation to optimize hyperparameters (39). This process was repeated 500 times with varying random seeds to capture performance stability across different patient subgroups. Model evaluation was conducted using accuracy, Brier class, and area under the ROC curve (AUC). Accuracy reflects the overall correctness of predictions, with values closer to 1 indicating better performance. The Brier score quantifies the disparity between anticipated probability and actual results, with lower scores signifying greater predictive accuracy (40). AUC quantifies the model’s ability to differentiate between positive and negative cases at varying thresholds, with higher values reflecting improved discriminatory power. AUC served as the primary metric for selecting the best-performing machine learning model alongside other performance indicators. For the top-performing model, the importance of various exposure factors and the model’s precision were further investigated. Statistical analyses were conducted using R software version 4.3.3, and a two-sided *p*-value of less than 0.05 was considered statistically significant.

## 3 Results

### 3.1 Characteristics of the study participants

Included in the analysis were 16,030 participants, with a weighted average age of 56.83 ± 10.94 years. The overall prevalence of COPD among participants was 11.9%, with a weighted mean LCD score of 11.63 ± 7.14. Following the execution of 1:1 PSM, the baseline characteristics of the groups were assessed utilizing standardized mean differences (SMD). Post-matching, all variables showed SMD values close to or below 0.1, meeting the statistical criteria for balance and indicating an optimal matching effect, as shown in Supplementary Figure 1. Furthermore, visual assessments through histograms and density plots demonstrated that the post-matching distributions between the groups were more similar, further confirming the balance of baseline characteristics, as presented in Supplementary Figure 2. Prior to matching, COPD patients were generally older, predominantly male, mostly non-Hispanic White, and had a higher smoking rate, as detailed in Table 1 (all *p* < 0.001). Additionally, the COPD group had lower education levels, a lower family income-to-poverty ratio, and lower BMI and WHtR, also shown in Table 1 (all *p* < 0.05). Following PSM, these differences were substantially reduced, and no significant differences were found between the COPD and non-COPD groups in terms of demographic characteristics, health behaviors, physical health indicators, and chronic diseases (all *p* > 0.05). Table 2 highlights that the COPD group had significantly lower LCD scores than the non-COPD group after matching (10.96 ± 7.02 vs. 12.09 ± 7.05, *p* < 0.001). Further analysis indicated that the COPD cohort had reduced consumption of fat, protein, and carbs relative to the non-COPD cohort. Supplementary Table 2 presents baseline characteristics of participants grouped by LCD score quartiles after matching.

**Table 1:** Weighted baseline characteristics of study participants stratified by COPD status, pre-PSM.

Characteristic	Overall (*n* = 16,030)	Non-COPD (*n* = 14,125)	COPD (*n* = 1,905)	*p*-value
Age (year)	56.83 ± (10.94)	56.32 ± (10.92)	60.44 ± (10.41)	<0.001
Sex (%)				0.002
Male	7,729 (47%)	6,673 (47%)	1,056 (53%)	
Female	8,301 (53%)	7,452 (53%)	849 (47%)	
Race/Ethnicity (%)				<0.001
Mexican American	1,941 (5.6%)	1,834 (6.1%)	107 (1.9%)	
Other Hispanic	1,593 (4.5%)	1,482 (4.9%)	111 (1.9%)	
Non-Hispanic White	7,410 (74%)	6,187 (73%)	1,223 (84%)	
Non-Hispanic Black	3,528 (9.8%)	3,165 (10%)	363 (7.0%)	
Other Race	1,558 (6.0%)	1,457 (6.1%)	101 (5.0%)	
Education level (%)				<0.001
< High school	3,303 (13%)	2,838 (12%)	465 (17%)	
High school	3,621 (23%)	3,079 (22%)	542 (29%)	
> High school	9,106 (64%)	8,208 (66%)	898 (54%)	
Marital status (%)				0.749
Unmarried	1,938 (10%)	1,707 (10%)	231 (10%)	
Married	14,092 (90%)	12,418 (90%)	1,674 (90%)	
PIR	3.33 ± (1.59)	3.37 ± (1.58)	3.06 ± (1.63)	<0.001
BMI (kg/m^2^)	29.42 ± (6.50)	29.56 ± (6.51)	28.45 ± (6.39)	<0.001
Waist circumference (cm)	101.53 ± (15.59)	101.54 ± (15.52)	101.44 ± (16.05)	0.475
WHtR	0.60 ± (0.09)	0.60 ± (0.09)	0.60 ± (0.09)	0.027
Physical activity (%)				0.081
Inactive	12,170 (75%)	10,677 (75%)	1,493 (78%)	
Active	3,860 (25%)	3,448 (25%)	412 (22%)	
Smoking status (%)				<0.001
No	8,502 (53%)	7,995 (57%)	507 (26%)	
Yes	7,528 (47%)	6,130 (43%)	1,398 (74%)	
Hypertension (%)				<0.001
No	8,708 (59%)	7,830 (61%)	878 (52%)	
Yes	7,322 (41%)	6,295 (39%)	1,027 (48%)	
Diabetes (%)				<0.001
No	13,160 (86%)	11,668 (87%)	1,492 (83%)	
Yes	2,870 (14%)	2,457 (13%)	413 (17%)	
Congestive heart failure (%)				<0.001
No	15,458 (97%)	13,729 (98%)	1,729 (93%)	
Yes	572 (2.5%)	396 (1.9%)	176 (6.9%)	
Coronary heart disease (%)				<0.001
No	15,204 (96%)	13,519 (96%)	1,685 (90%)	
Yes	826 (4.4%)	606 (3.7%)	220 (9.7%)	
Heart disease (%)				<0.001
No	15,248 (96%)	13,549 (97%)	1,699 (92%)	
Yes	782 (3.8%)	576 (3.2%)	206 (8.4%)	
Stroke (%)				<0.001
No	15,313 (97%)	13,544 (97%)	1,769 (94%)	
Yes	717 (3.3%)	581 (3.0%)	136 (6.1%)	
Magnesium intake (mg)	306.18 ± (127.34)	307.24 ± (126.51)	298.61 ± (132.87)	0.010
Calcium intake (mg)	939.88 ± (473.15)	940.61 ± (468.44)	934.65 ± (505.47)	0.187
Vitamin D intake (mcg)	4.77 ± (4.61)	4.75 ± (4.58)	4.90 ± (4.85)	0.498
Fat intake score	4.99 ± (3.92)	5.04 ± (3.92)	4.68 ± (3.94)	0.005
Protein intake score	2.72 ± (3.13)	2.78 ± (3.15)	2.36 ± (3.02)	<0.001
Carbohydrate intake score	3.91 ± (3.12)	3.92 ± (3.12)	3.91 ± (3.17)	0.699
LCD score	11.63 ± (7.14)	11.73 ± (7.15)	10.95 ± (7.01)	0.003

Mean ± SD for continuous variables: *p* values were calculated using weighted Student’s *t-*tests. For categorical variables, n (%), *p* values were calculated using weighted Chi-square tests. PIR, poverty-to-income ratio; BMI, body mass index; WHtR, waist-to-height ratio; LCD score, low-carbohydrate-diet score; COPD, chronic obstructive pulmonary disease.

**Table 2:** Weighted baseline characteristics of study participants stratified by COPD status, post-PSM.

Characteristic	Overall(*n* = 3,764)	Non-COPD(*n* = 1,882)	COPD(*n* = 1,882)	*p*-value
Age (year)	60.23 ± (10.85)	60.12 ± (11.29)	60.35 ± (10.38)	0.595
Sex (%)				0.582
Male	2,104 (53%)	1,067 (54%)	1,037 (52%)	
Female	1,660 (47%)	815 (46%)	845 (48%)	
Race/Ethnicity (%)				0.511
Mexican American	220 (2.1%)	113 (2.3%)	107 (2.0%)	
Other Hispanic	211 (2.0%)	100 (2.2%)	111 (1.9%)	
Non-Hispanic White	2,355 (84%)	1,155 (83%)	1,200 (84%)	
Non-Hispanic Black	690 (6.8%)	327 (6.6%)	363 (7.0%)	
Other Race	288 (5.5%)	187 (6.0%)	101 (5.0%)	
Education level (%)				0.236
< High school	894 (16%)	438 (16%)	456 (17%)	
High school	1,051 (28%)	520 (26%)	531 (29%)	
> High school	1,819 (56%)	924 (58%)	895 (55%)	
Marital status (%)				0.975
Unmarried	3,329 (90%)	1,674 (90%)	1,655 (90%)	
Married	435 (10%)	208 (10%)	227 (10%)	
PIR	3.08 ± (1.63)	3.08 ± (1.62)	3.07 ± (1.63)	0.919
BMI (kg/m^2^)	28.55 ± (6.13)	28.65 ± (5.86)	28.45 ± (6.39)	0.121
Waist circumference (cm)	101.42 ± (15.48)	101.42 ± (14.87)	101.43 ± (16.07)	0.581
WHtR	0.60 ± (0.09)	0.60 ± (0.09)	0.60 ± (0.09)	0.376
Physical activity (%)				0.520
Inactive	2,959 (78%)	1,486 (79%)	1,473 (78%)	
Active	805 (22%)	396 (21%)	409 (22%)	
Smoking status (%)				0.402
No	1,008 (27%)	501 (28%)	507 (27%)	
Yes	2,756 (73%)	1,381 (72%)	1,375 (73%)	
Hypertension (%)				0.752
No	1,769 (52%)	893 (53%)	876 (52%)	
Yes	1,995 (48%)	989 (47%)	1,006 (48%)	
Diabetes (%)				0.617
No	2,952 (83%)	1,475 (84%)	1,477 (83%)	
Yes	812 (17%)	407 (16%)	405 (17%)	
Congestive heart failure (%)				0.587
No	3,461 (94%)	1,734 (94%)	1,727 (94%)	
Yes	303 (5.8%)	148 (5.6%)	155 (6.1%)	
Coronary heart disease (%)				0.492
No	3,376 (91%)	1,696 (92%)	1,680 (91%)	
Yes	388 (8.8%)	186 (8.5%)	202 (9.2%)	
Heart disease (%)				0.757
No	3,399 (92%)	1,704 (93%)	1,695 (92%)	
Yes	365 (7.6%)	178 (7.4%)	187 (7.8%)	
Stroke (%)				0.706
No	3,497 (94%)	1,745 (95%)	1,752 (94%)	
Yes	267 (5.7%)	137 (5.5%)	130 (5.8%)	
Magnesium intake (mg)	298.41 ± (128.27)	298.00 ± (123.18)	298.82 ± (133.24)	0.695
Calcium intake (mg)	934.73 ± (514.88)	935.15 ± (524.25)	934.31 ± (505.40)	0.990
Vitamin D intake (mcg)	4.93 ± (5.26)	4.97 ± (5.65)	4.88 ± (4.84)	0.631
Fat intake score	4.96 ± (3.91)	5.23 ± (3.87)	4.68 ± (3.94)	0.002
Protein intake score	2.55 ± (3.10)	2.73 ± (3.17)	2.37 ± (3.02)	0.010
Carbohydrate intake score	4.02 ± (3.16)	4.13 ± (3.14)	3.92 ± (3.18)	0.045
LCD score	11.53 ± (7.06)	12.09 ± (7.05)	10.96 ± (7.02)	<0.001

Mean ± SD for continuous variables: *p* values were calculated using weighted Student’s *t*-tests. For categorical variables, n (%), *p* values were calculated using weighted Chi-square tests. PIR, poverty-to-income ratio; BMI, body mass index; WHtR, waist-to-height ratio; LCD score, low-carbohydrate-diet score; COPD, chronic obstructive pulmonary disease.

### 3.2 Association between LCD score and COPD

**Figure 2:**
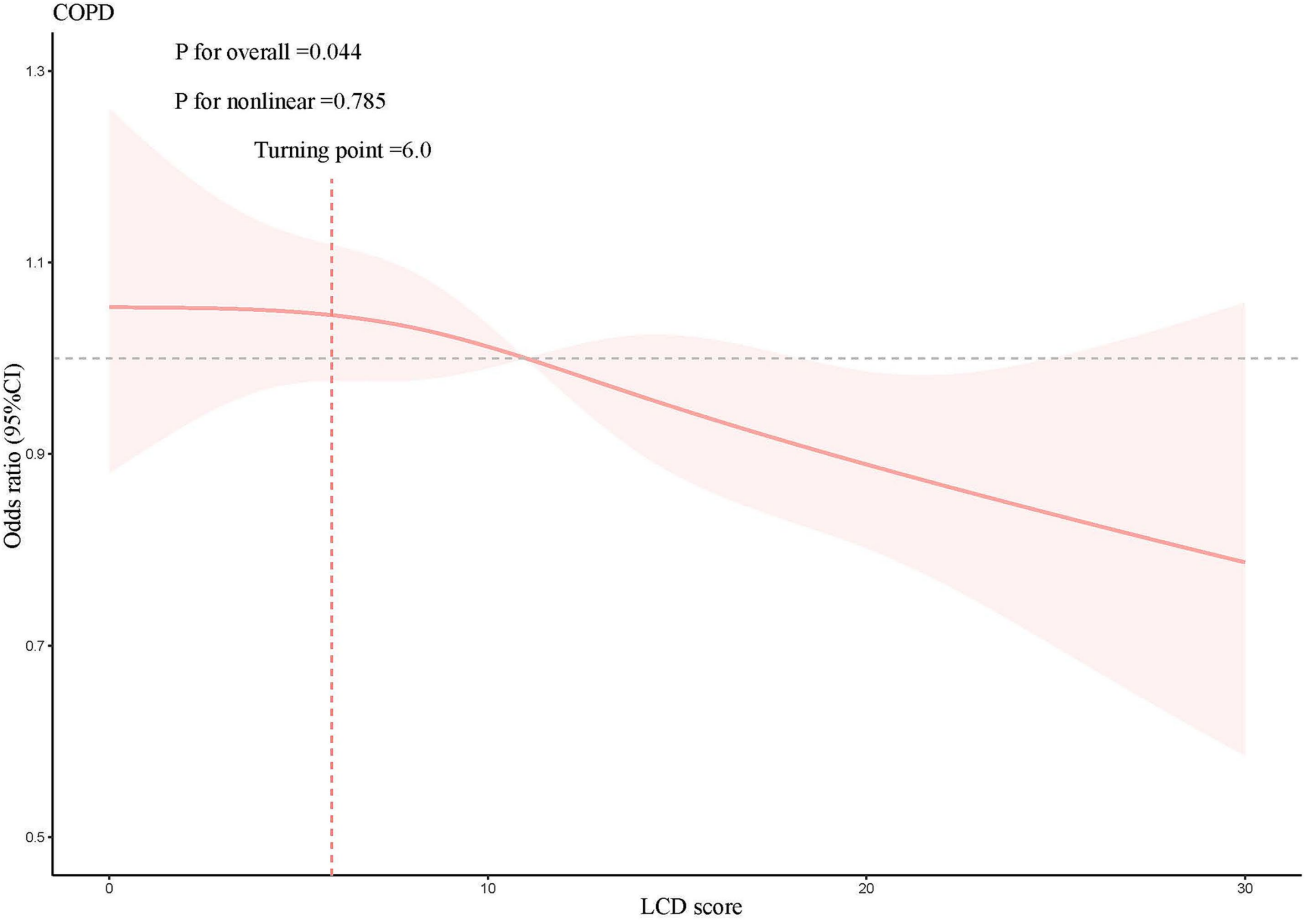
Results of the RCS analysis, adjusted for age, sex, race/ethnicity, educational level, marital status, PIR, BMI, waist circumference, WHtR, physical activity, smoking status, hypertension, diabetes, congestive heart failure, coronary heart disease, heart disease, stroke, magnesium intake, calcium intake, vitamin D intake.

**Figure 3:**
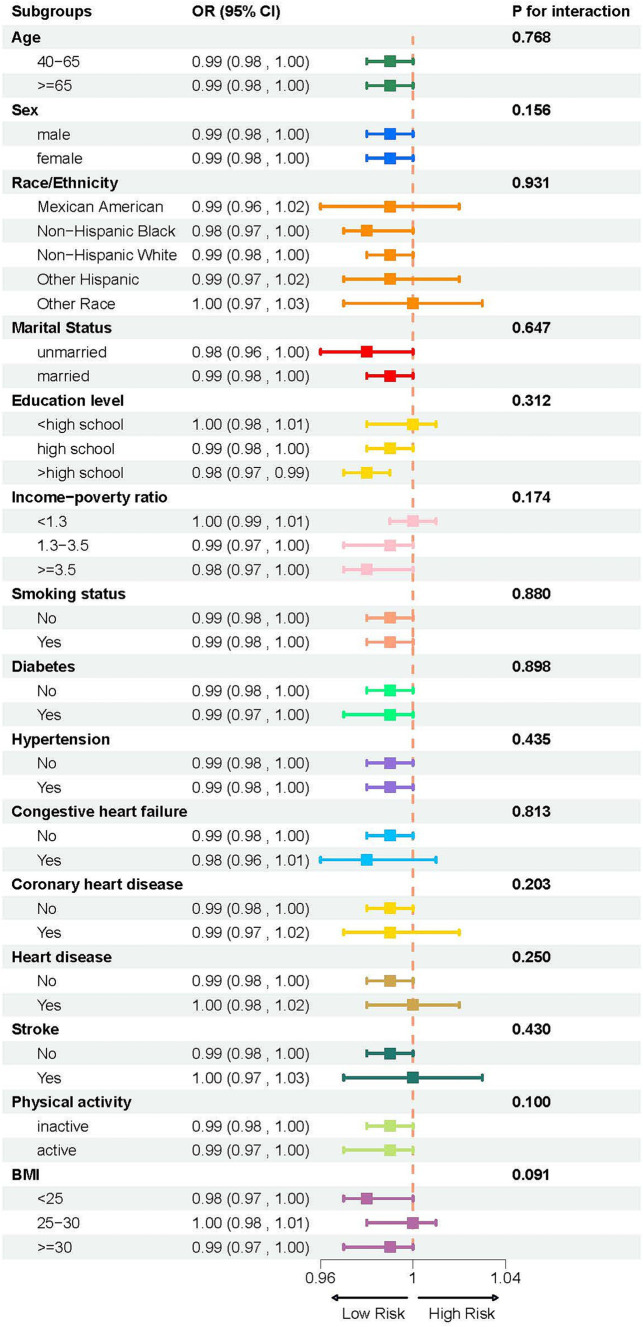
Subgroup analysis of the association between LCD score and COPD, stratified by age (40–65 years, ≥65 years), sex (male, female), race/ethnicity (Mexican American, other Hispanic, non-Hispanic White, non-Hispanic Black, other races), marital status (unmarried, married), educational level (< high school, high school, > high school), PIR (<1.3, 1.3–3.5, ≥3.5), smoking status (non-smoker, smoker), diabetes (no, yes), hypertension (no, yes), congestive heart failure (no, yes), coronary heart disease (no, yes), heart disease (no, yes), stroke (no, yes), physical activity (inactive, active), and BMI (<25, 25–30, and ≥ 30).

A weighted multivariate logistic regression was performed to analyze the relationship between the LCD score and COPD, as illustrated in Table 3. The analysis indicated that higher LCD scores were significantly associated with lower odds of COPD prevalence. Subsequent to the adjustment for possible confounders, the adjusted ORs with 95% CIs for COPD across the higher quartiles of the LCD score, compared to the lowest quartile, were 0.77 (0.63, 0.95), 0.74 (0.59, 0.93), and 0.61 (0.48, 0.78), respectively. Additionally, an RCS curve (Figure 2) revealed a linear inverse association between the LCD score and the odds of COPD prevalence, with a notable reduction in the odds of COPD prevalence once the LCD score exceeded 6.0. A stratified a9nalysis further assessed the consistency of this association across various subgroups. As illustrated in Figure 3, none of the stratification variables—including age (40–65 years, ≥65 years), sex (male, female), race/ethnicity (Mexican American, other Hispanic, non-Hispanic White, non-Hispanic Black, other races), marital status (unmarried, married), educational level (< high school, high school, > high school), PIR (<1.3, 1.3–3.5, ≥3.5), smoking status (non-smoker, smoker), diabetes (no, yes), hypertension (no, yes), congestive heart failure (no, yes), coronary heart disease (no, yes), heart disease (no, yes), stroke (no, yes), physical activity (inactive, active), and BMI (normal weight, overweight, obese) (41)—significantly modified the association between the LCD score and the odds of COPD prevalence (*P* for interaction >0.05).

**Table 3:** Weighted logistic regression analysis of the association between LCD score and COPD.

	Crude model OR (95%CI) *p*-value	Model 1 OR (95%CI) *p*-value	Model 2 OR (95%CI) *p*-value
LCD score	0.98 (0.97, 0.99) <0.001	0.98 (0.97, 0.99) <0.001	0.98 (0.96, 0.99) <0.001
LCD score quartile			
Low	Ref.	Ref.	Ref.
Lower Middle	0.78 (0.63, 0.96) 0.020	0.78 (0.63, 0.96) 0.018	0.77 (0.63, 0.95) 0.015
Upper Middle	0.75 (0.59, 0.94) 0.012	0.74 (0.59, 0.93) 0.009	0.74 (0.59, 0.93) 0.010
High	0.63 (0.50, 0.80) <0.001	0.63 (0.50, 0.79) <0.001	0.61 (0.48, 0.78) <0.001
*P* for trend	<0.001	<0.001	<0.001

Data are presented as OR (95% CI). Crude model: Did not adjust for any potential confounders; Model 1: Adjusted for age, sex, race/ethnicity, educational level, marital status, PIR; Model 2: Adjusted for age, sex, race/ethnicity, PIR, marital status, educational level, BMI, waist circumference, WHtR, physical activity, smoking status, hypertension, diabetes, congestive heart failure, coronary heart disease, heart disease, stroke, magnesium intake, calcium intake, vitamin D intake; Quantiles: Low (1st quantile), Lower Middle (2nd quantile), Upper Middle (3rd quantile), High (4th quantile).

### 3.3 Machine learning model performance and validation

**Figure 4:**
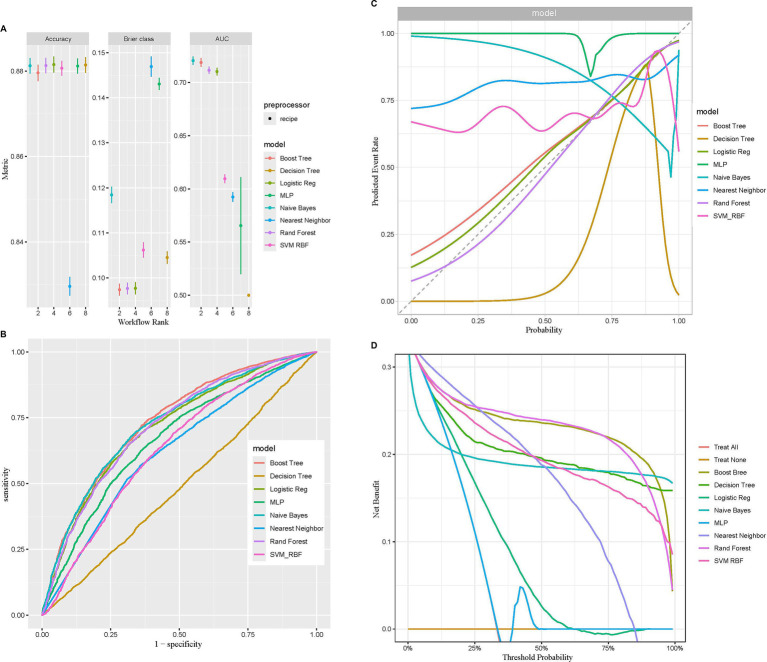
Comparison of eight machine learning models in terms of predictive performance. **(A)** Performance comparison based on accuracy, Brier class, and AUC, highlighting predictive accuracy and reliability. **(B)** ROC curves illustrating the discriminative ability of each model. **(C)** Calibration curves assessing the agreement between predicted probabilities and observed outcomes for the eight models. **(D)** DCA evaluating the clinical utility of each model across a range of threshold probabilities.

Machine learning represents a sophisticated approach to pattern recognition, allowing machines to draw conclusions by processing extensive datasets (42). The predicted efficacy of diverse machine learning models was evaluated using metrics like accuracy, Brier score, and AUC. The random forest model attained the maximum accuracy, the lowest Brier score, and an AUC value of 0.713, positioning it among the top three models (Figure 4A). Moreover, it demonstrated superior performance on the ROC and DCA curves compared to others, indicating both strong predictive performance and clinical relevance (Figures 4B,D). The calibration curve was close to the diagonal line, suggesting the model is well-calibrated and does not exhibit significant overfitting (Figure 4C). Thus, based on these performance evaluation metrics, the random forest model displayed the best, nearly perfect predictive capability.

**Figure 5:**
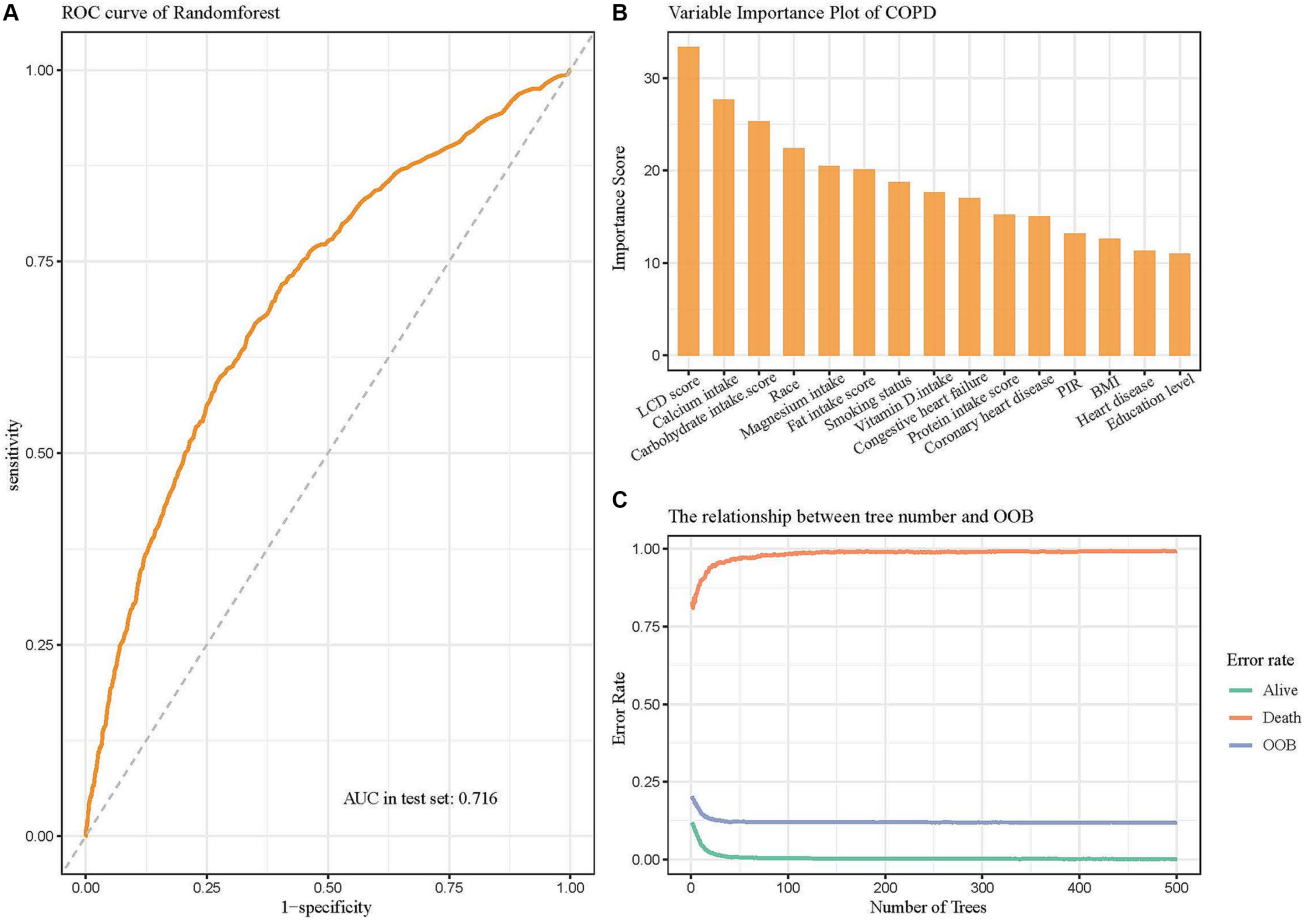
Random forest model evaluating the significance of the LCD score in predicting COPD. **(A)** ROC curve of the model after hyperparameter optimization. **(B)** Variable importance plot showing the contributions of different predictors. **(C)** The relationship between the number of decision trees and OOB error rate.

After the random forest model was selected, the data were partitioned into training and validation sets, with 70% allocated to the training set and 30% to the validation set. The training set was used to analyze independent risk factors, perform importance ranking, and construct a regression equation. Internal validation was performed using the original dataset as the test set, with the ROC curve demonstrating an area under the curve (AUC) of 0.716, indicating good discrimination and predictive ability (Figure 5A). Among the variable importance rankings, the LCD score made a significant contribution to the predictive model (Figure 5B). To further evaluate model performance and convergence during training, the OOB classification error rate curve was plotted. The curve showed a gradual decrease in error rate as the number of decision trees increased, eventually stabilizing, indicating that the model reached a relatively stable state (Figure 5C).

## 4 Discussion

In a study involving 16,030 NHANES participants, we applied PSM to minimize group differences by matching participants with similar key characteristics, ensuring balanced baseline features between COPD and non-COPD groups. We found that the average LCD score of COPD patients was significantly lower than that of non-COPD patients, further supporting the linear negative correlation between the LCD score and COPD, which is not influenced by various confounding factors. Subsequent subgroup analyses confirmed that this correlation remained stable across different groups. Utilizing community data, we collected information through interviews and employed eight machine learning methods (including BT, DT, LR, MLP, NB, KNN, RF, and SVM-RBF) to construct predictive models. After conducting discrimination, fitting, and clinical efficacy assessments, we determined that the random forest model is the most efficacious for assessing the correlation between the LCD score and the odds of COPD prevalence, demonstrating strong predictive capability. Our findings underscore the significance of low carbohydrate intake in reducing the odds of COPD prevalence.

Current data underscores the vital importance of dietary nutrition in the onset and advancement of respiratory illnesses. Consequently, a growing body of research has started exploring how dietary patterns and nutritional factors influence the prevention and treatment of COPD. A meta-analysis conducted by Zheng PF et al. (43) indicates that unhealthy dietary patterns, particularly high intakes of red meat, processed meats, refined grains, sweets, desserts, and fried potatoes, correlate with a heightened risk of developing COPD. Such high-carbohydrate diets may lead to excessive carbon dioxide production, thereby increasing the respiratory burden (44–47). A three-week controlled trial involving 60 COPD patients revealed that the low-carbohydrate group exhibited a modest yet statistically significant increase in forced expiratory volume in 1 s (FEV1) when compared with the high-carbohydrate group. Additionally, Ricciardolo FL et al. (48) found that the high concentrations of nitrates, nitrites, and nitrosamines in cured and processed meats can generate reactive nitrogen species in the body, further exacerbating airway and lung inflammation, leading to DNA damage and mitochondrial respiratory inhibition, which may contribute to the gradual deterioration of lung function. Clinical studies by Walter RE et al. (49) and Cazzola M et al. (50) have shown a significant association between high glycemic index foods, such as refined grains and desserts, and impaired lung function, with lung function impairment being a critical diagnostic criterion for COPD.

The influence of dietary patterns on COPD is a significant field of research, especially on the contribution of high-carbohydrate diets to elevated carbon dioxide production, which aggravates the respiratory burden in patients. Carbohydrates have a RQ of 1.0, meaning that for every unit of oxygen consumed, an equal amount of carbon dioxide is produced. In contrast, the RQ of fats is approximately 0.7, indicating that fat metabolism produces less carbon dioxide. Therefore, a diet rich in carbohydrates may result in elevated carbon dioxide generation, thereby intensifying the respiratory load in COPD patients (44, 46, 47). Clinical studies have confirmed this hypothesis. Research shows that COPD patients consuming a high-carbohydrate diet exhibit a significant increase in carbon dioxide production (VCO2) and respiratory rate, particularly within 30 to 60 min post-meal, with effects lasting up to 1.5 h (45, 47). Moreover, patients experience a marked increase in perceived breathlessness during physical activity (46). These findings suggest that high-carbohydrate diets not only affect basal metabolism but also directly worsen the respiratory burden in COPD patients. The increased carbon dioxide production significantly intensifies breathlessness, and for patients with impaired lung function, this additional burden may worsen discomfort and reduce exercise tolerance. For example, one study found that after consuming a high-carbohydrate meal, the VCO2 in COPD patients increased from 0.23 L/min to 0.29 L/min, and minute ventilation increased from 10.3 L/min to 12.8 L/min (44, 46). This change highlights the significant impact of a high-carbohydrate diet on the respiratory system and underscores the importance of dietary management in COPD patients, particularly reducing carbohydrate intake to decrease carbon dioxide production and alleviate respiratory burden.

As a specialized dietary intervention, LCD has been shown to improve respiratory function. The LCD score, by offering a more quantitative and personalized assessment, enables a more accurate evaluation of an individual’s response to dietary changes, thus further enhancing the benefits for respiratory function. Increasing the intake of fats and proteins while reducing carbohydrates can not only alleviate the burden on pulmonary ventilation but also suppress insulin secretion, contributing to better regulation of glucose and lipid metabolism (24). Although carbohydrates remain an essential nutrient, limiting their intake and choosing fiber-rich sources, such as millet and oats, is advisable to ensure balanced nutrition. Moreover, high-fat meals ought to emphasize unsaturated fatty acids present in plant-derived oils, such as tea and olive oil, while minimizing excessive animal fats to mitigate the risk of cardiovascular disease (51, 52). Notably, while some patients can tolerate the increased carbon dioxide load caused by a high-carbohydrate diet, this burden can significantly worsen symptoms in individuals with severe pulmonary diseases. Therefore, it is crucial to develop personalized dietary plans tailored to each patient’s specific clinical condition and metabolic profile (44).

This study investigates the correlation between LCD scores and the odds of COPD prevalence in the US population utilizing data from the NHANES database. The findings indicate a possible correlation between reduced carbohydrate intake and lower odds of developing COPD, offering potential guidance for dietary interventions. By comparing eight machine learning algorithms, we identified the most effective model for predicting patients associated with odds of COPD prevalence. This model offers a practical method for early identification of individuals susceptible to COPD, facilitating the creation of targeted prevention and intervention strategies. A principal strength of our study lies in the use of a multi-stage probability sampling approach, which improves the representativeness and reliability of the results.

However, this study possesses specific limitations. First, the majority of the predictors utilized in our research were derived from self-reported data from individuals, potentially introducing bias. Nevertheless, the NHANES database employs a highly standardized data collection process, and the large sample size in our study helps to mitigate this bias to some extent. Second, although we conducted internal validation by dividing the research data set into training and validation subsets, we lacked an external cohort to further assess the model’s performance. Additionally, given that the study population was exclusively from the United States, caution is warranted when extrapolating these findings to other groups, as factors such as racial differences and geographic location may influence the results. Future research should focus on validating these results through the use of external datasets, particularly from different continents, to ensure broader applicability and robustness of the model.

## 5 Conclusion

In summary, this study highlights a significant relationship between LCD scores and the prevalence of COPD among American adults. The machine learning model developed using the random forest method showed solid predictive performance. Nonetheless, additional prospective research and randomized controlled trials are essential to corroborate these findings, investigate underlying mechanisms, and assess potential treatment implications.

## Data Availability

The datasets presented in this study can be found in online repositories. The names of the repository/repositories and accession number(s) can be found in the article/Supplementary material.

## Glossary

LCD Score: Low Carbohydrate Diet Score
COPD: Chronic Obstructive Pulmonary Disease
RQ: Respiratory Quotient
CO₂: Carbon Dioxide
VCO₂: Carbon Dioxide Production
FEV1: Forced Expiratory Volume in 1 s
FVC - Forced Vital Capacity
PIR: Poverty-to-Income Ratio
BMI: Body Mass Index
WHtR: Waist-to-Height Ratio
DM: Diabetes Mellitus
CVD: Cardiovascular Disease
OGTT: Oral Glucose Tolerance Test
NHANES: National Health and Nutrition Examination Survey
NCHS: National Center for Health Statistics
CDC: Centers for Disease Control and Prevention
MEC: Mobile Examination Center
FNDDS: Food and Nutrition Database for Dietary Studies
GPAQ: Global Physical Activity Questionnaire
QA: Quality Assurance
QC: Quality Control
SP: Sample Person
PSM: Propensity Score Matching
RCS Curve: Restricted Cubic Splines Curve
ROC Curve: Receiver Operating Characteristic Curve
DCA: Decision Curve Analysis
AUC: Area Under the Curve
SD: Standard Deviation
IQR: Interquartile Range
OR: Odds Ratio
CI: Confidence Interval
MLP: Multilayer Perceptron
KNN: K-Nearest Neighbors
SVM-RBF: Support Vector Machine with a Radial Basis Function

## References

[1] Agustí A Celli BR Criner GJ Halpin D Anzueto A Barnes P. Global initiative for chronic obstructive lung disease 2023 report: gold executive summary. Arch Bronconeumol. (2023) 59:232–48. doi: 10.1016/j.arbres.2023.02.009, PMID: 36933949

[2] Christenson SA Smith BM Bafadhel M Putcha N. Chronic obstructive pulmonary disease. Lancet. (2022) 399:2227–42. doi: 10.1016/s0140-6736(22)00470-6 35533707

[3] Chen S Kuhn M Prettner K Yu F Yang T Bärnighausen T. The global economic burden of chronic obstructive pulmonary disease for 204 countries and territories in 2020-50: a health-augmented macroeconomic modelling study. Lancet Glob Health. (2023) 11:e1183–93. doi: 10.1016/s2214-109x(23)00217-6, PMID: 37474226 PMC10369014

[4] Vos T Lim SS Abbafati C Abbas KM Abbasi M Abbasifard M. Global burden of 369 diseases and injuries in 204 countries and territories, 1990-2019: a systematic analysis for the global burden of disease study 2019 Lancet. (2020) 396:1204–22. doi: 10.1016/s0140-6736(20)30925-9, PMID: 33069326 PMC7567026

[5] Czarnecka-Chrebelska KH Mukherjee D Maryanchik SV Rudzinska-Radecka M. Biological and genetic mechanisms of Copd, its diagnosis, treatment, and relationship with lung Cancer. Biomedicine. (2023) 11:448. doi: 10.3390/biomedicines11020448, PMID: 36830984 PMC9953173

[6] Lamprecht B McBurnie MA Vollmer WM Gudmundsson G Welte T Nizankowska-Mogilnicka E. Copd in never smokers: results from the population-based burden of obstructive lung disease study. Chest. (2011) 139:752–63. doi: 10.1378/chest.10-1253, PMID: 20884729 PMC3168866

[7] Yang IA Jenkins CR Salvi SS. Chronic obstructive pulmonary disease in never-smokers: risk factors, pathogenesis, and implications for prevention and treatment. Lancet Respir Med. (2022) 10:497–511. doi: 10.1016/s2213-2600(21)00506-3, PMID: 35427530

[8] Beijers R Steiner MC Schols A. The role of diet and nutrition in the Management of Copd. Eur Respir Rev. (2023) 32:230003. doi: 10.1183/16000617.0003-2023, PMID: 37286221 PMC10245132

[9] Heefner A Simovic T Mize K Rodriguez-Miguelez P. The role of nutrition in the development and Management of Chronic Obstructive Pulmonary Disease. Nutrients. (2024) 16:1136. doi: 10.3390/nu16081136, PMID: 38674827 PMC11053888

[10] Tian TL Zhi TY Xie ML Jiang YL Qu XK. Dietary inflammatory index and all-cause mortality in adults with Copd: a prospective cohort study from the Nhanes 1999-2018. Front Nutr. (2024) 11:1421450. doi: 10.3389/fnut.2024.1421450, PMID: 39385783 PMC11463153

[11] Halton TL Willett WC Liu S Manson JE Albert CM Rexrode K. Low-carbohydrate-diet score and the risk of coronary heart disease in women. N Engl J Med. (2006) 355:1991–2002. doi: 10.1056/NEJMoa055317, PMID: 17093250

[12] Scoditti E Massaro M Garbarino S Toraldo DM. Role of diet in chronic obstructive pulmonary disease prevention and treatment. Nutrients. (2019) 11:1357. doi: 10.3390/nu11061357, PMID: 31208151 PMC6627281

[13] McClave SA Lowen CC Kleber MJ McConnell JW Jung LY Goldsmith LJ. Clinical use of the respiratory quotient obtained from indirect calorimetry. JPEN J Parenter Enteral Nutr. (2003) 27:21–6. doi: 10.1177/014860710302700121 12549594

[14] Ha K Kim K Chun OK Joung H Song Y. Differential Association of Dietary Carbohydrate Intake with metabolic syndrome in the us and Korean adults: data from the 2007-2012 Nhanes and Knhanes. Eur J Clin Nutr. (2018) 72:848–60. doi: 10.1038/s41430-017-0031-8, PMID: 29339830

[15] Vogelmeier CF Criner GJ Martínez FJ Anzueto A Barnes PJ Bourbeau J. Erratum to global strategy for the diagnosis, management, and prevention of chronic obstructive lung disease 2017 report: gold executive summary. Arch Bronconeumol. (2017) 53:411–2. doi: 10.1016/j.arbres.2017.06.001, PMID: 28668138

[16] Wylie-Rosett J Aebersold K Conlon B Isasi CR Ostrovsky NW. Health effects of low-carbohydrate diets: where should new research go? Curr Diab Rep. (2013) 13:271–8. doi: 10.1007/s11892-012-0357-5, PMID: 23266565 PMC3595318

[17] Cai B Zhu Y Ma Y Xu Z Zao Y Wang J. Effect of supplementing a high-fat, low-carbohydrate enteral formula in Copd patients. Nutrition. (2003) 19:229–32. doi: 10.1016/s0899-9007(02)01064-x, PMID: 12620524

[18] Hsieh MJ Yang TM Tsai YH. Nutritional supplementation in patients with chronic obstructive pulmonary disease. J Formos Med Assoc. (2016) 115:595–601. doi: 10.1016/j.jfma.2015.10.008 26822811

[19] Frankfort JD Fischer CE Stansbury DW McArthur DL Brown SE Light RW. Effects of high-and low-carbohydrate meals on maximum exercise performance in chronic airflow obstruction. Chest. (1991) 100:792–5. doi: 10.1378/chest.100.3.792, PMID: 1889274

[20] Nanri A Mizoue T Kurotani K Goto A Oba S Noda M. Low-carbohydrate diet and type 2 diabetes risk in Japanese men and women: the Japan public health center-based prospective study. PLoS One. (2015) 10:e0118377. doi: 10.1371/journal.pone.0118377, PMID: 25695497 PMC4335023

[21] Sangsefidi ZS Lorzadeh E Nadjarzadeh A Mirzaei M Hosseinzadeh M. The association between low-carbohydrate diet score and metabolic syndrome among Iranian adults. Public Health Nutr. (2021) 24:6299–308. doi: 10.1017/s1368980021003074, PMID: 34294177 PMC11148582

[22] Farhadnejad H Asghari G Teymoori F Tahmasebinejad Z Mirmiran P Azizi F. Low-carbohydrate diet and cardiovascular diseases in Iranian population: Tehran lipid and glucose study. Nutr Metab Cardiovasc Dis. (2020) 30:581–8. doi: 10.1016/j.numecd.2019.11.012, PMID: 32008914

[23] Wang H Lv Y Ti G Ren G. Association of low-Carbohydrate-Diet Score and Cognitive Performance in older adults: National Health and nutrition examination survey (Nhanes). BMC Geriatr. (2022) 22:983. doi: 10.1186/s12877-022-03607-1, PMID: 36539697 PMC9764565

[24] Malmir H Onvani S Ardestani ME Feizi A Azadbakht L Esmaillzadeh A. Adherence to low carbohydrate diet in relation to chronic obstructive pulmonary disease. Front Nutr. (2021) 8:690880. doi: 10.3389/fnut.2021.690880, PMID: 34414207 PMC8368978

[25] Min Y Wei X Wei Z Song G Zhao X Lei Y. Prognostic effect of triglyceride glucose-related parameters on all-cause and cardiovascular mortality in the United States adults with metabolic dysfunction-associated Steatotic liver disease. Cardiovasc Diabetol. (2024) 23:188. doi: 10.1186/s12933-024-02287-y, PMID: 38824550 PMC11144336

[26] Zhang X Liang J Luo H Zhang H Xiang J Guo L. The association between body roundness index and osteoporosis in American adults: analysis from Nhanes dataset. Front Nutr. (2024) 11:1461540. doi: 10.3389/fnut.2024.1461540, PMID: 39430785 PMC11486732

[27] Mazidi M Katsiki N Mikhailidis DP Sattar N Banach M. Lower carbohydrate diets and all-cause and cause-specific mortality: a population-based cohort study and pooling of prospective studies. Eur Heart J. (2019) 40:2870–9. doi: 10.1093/eurheartj/ehz174, PMID: 31004146

[28] Ahluwalia N Dwyer J Terry A Moshfegh A Johnson C. Update on Nhanes dietary data: focus on collection, release, analytical considerations, and uses to inform public policy. Adv Nutr. (2016) 7:121–34. doi: 10.3945/an.115.009258, PMID: 26773020 PMC4717880

[29] Wang X Wen J Gu S Zhang L Qi X. Frailty in asthma-Copd overlap: a cross-sectional study of association and risk factors in the Nhanes database. BMJ Open Respir Res. (2023) 10:e001713. doi: 10.1136/bmjresp-2023-001713, PMID: 37336621 PMC10314668

[30] Xu Y Yan Z Li K Liu L Xu L. Association between nutrition-related indicators with the risk of chronic obstructive pulmonary disease and all-cause mortality in the elderly population: evidence from Nhanes. Front Nutr. (2024) 11:1380791. doi: 10.3389/fnut.2024.1380791, PMID: 39081677 PMC11286481

[31] Benedetto U Head SJ Angelini GD Blackstone EH. Statistical primer: propensity score matching and its alternatives. Eur J Cardiothorac Surg. (2018) 53:1112–7. doi: 10.1093/ejcts/ezy167, PMID: 29684154

[32] Kane LT Fang T Galetta MS Goyal DKC Nicholson KJ Kepler CK. Propensity score matching: a statistical method. Clin Spine Surg. (2020) 33:120–2. doi: 10.1097/bsd.0000000000000932 31913173

[33] Lenis D Nguyen TQ Dong N Stuart EA. It's all about balance: propensity score matching in the context of complex survey data. Biostatistics. (2019) 20:147–63. doi: 10.1093/biostatistics/kxx063, PMID: 29293896 PMC6296318

[34] Chen TC Parker JD Clark J Shin HC Rammon JR Burt VL. National Health and nutrition examination survey: estimation procedures, 2011-2014. Vital Health Stat 2. (2018) 177:1–26. PMID: 29775431

[35] Chen TC Clark J Riddles MK Mohadjer LK Fakhouri THI. National Health and nutrition examination survey, 2015-2018: sample design and estimation procedures. Vital Health Stat. (2020) 2:1–35. 33663649

[36] Liu Y Li K Li C Feng Z Cai Y Zhang Y. Pesticides, Cancer, and oxidative stress: an application of machine learning to Nhanes data. Environ Sci Eur. (2024) 36:8. doi: 10.1186/s12302-023-00834-0

[37] Guo J He Q Li Y. Machine learning-based prediction of vitamin D deficiency: Nhanes 2001-2018. Front Endocrinol (Lausanne). (2024) 15:1327058. doi: 10.3389/fendo.2024.1327058, PMID: 38449846 PMC10916299

[38] Huang AA Huang SY. Increasing transparency in machine learning through bootstrap simulation and shapely additive explanations. PLoS One. (2023) 18:e0281922. doi: 10.1371/journal.pone.0281922, PMID: 36821544 PMC9949629

[39] Khalilia M Chakraborty S Popescu M. Predicting disease risks from highly imbalanced data using random Forest. BMC Med Inform Decis Mak. (2011) 11:51. doi: 10.1186/1472-6947-11-51, PMID: 21801360 PMC3163175

[40] Huang AA Huang SY. Computation of the distribution of model accuracy statistics in machine learning: comparison between analytically derived distributions and simulation-based methods. Health Sci Rep. (2023) 6:e1214. doi: 10.1002/hsr2.1214, PMID: 37091362 PMC10119581

[41] Curry SJ Krist AH Owens DK Barry MJ Caughey AB Davidson KW. Behavioral weight loss interventions to prevent obesity-related morbidity and mortality in adults: us preventive services task force recommendation statement. JAMA. (2018) 320:1163–71. doi: 10.1001/jama.2018.13022, PMID: 30326502

[42] Vollmer A Vollmer M Lang G Straub A Shavlokhova V Kübler A. Associations between periodontitis and Copd: an artificial intelligence-based analysis of Nhanes iii. J Clin Med. (2022) 11:7210. doi: 10.3390/jcm11237210, PMID: 36498784 PMC9737076

[43] Zheng PF Shu L Si CJ Zhang XY Yu XL Gao W. Dietary patterns and chronic obstructive pulmonary disease: a Meta-analysis. COPD. (2016) 13:515–22. doi: 10.3109/15412555.2015.1098606, PMID: 26678388

[44] Gieseke T Gurushanthaiah G Glauser FL. Effects of carbohydrates on carbon dioxide excretion in patients with airway disease. Chest. (1977) 71:55–8. doi: 10.1378/chest.71.1.55, PMID: 830500

[45] Askanazi J Rosenbaum SH Hyman AI Silverberg PA Milic-Emili J Kinney JM. Respiratory changes induced by the large glucose loads of Total parenteral nutrition. JAMA. (1980) 243:1444–7. doi: 10.1001/jama.1980.03300400028023, PMID: 6767043

[46] Efthimiou J Mounsey PJ Benson DN Madgwick R Coles SJ Benson MK. Effect of carbohydrate rich versus fat rich loads on gas exchange and walking performance in patients with chronic obstructive lung disease. Thorax. (1992) 47:451–6. doi: 10.1136/thx.47.6.451, PMID: 1496505 PMC463811

[47] Kuo CD Shiao GM Lee JD. The effects of high-fat and high-carbohydrate diet loads on gas exchange and ventilation in Copd patients and Normal subjects. Chest. (1993) 104:189–96. doi: 10.1378/chest.104.1.189, PMID: 8325067

[48] Ricciardolo FL Di Stefano A Sabatini F Folkerts G. Reactive nitrogen species in the respiratory tract. Eur J Pharmacol. (2006) 533:240–52. doi: 10.1016/j.ejphar.2005.12.057 16464450

[49] Walter RE Beiser A Givelber RJ O'Connor GT Gottlieb DJ. Association between glycemic state and lung function: the Framingham heart study. Am J Respir Crit Care Med. (2003) 167:911–6. doi: 10.1164/rccm.2203022 12623860

[50] Cazzola M Rogliani P Ora J Calzetta L Lauro D Matera MG. Hyperglycaemia and chronic obstructive pulmonary disease. Diagnostics (Basel). (2023) 13:3362. doi: 10.3390/diagnostics13213362, PMID: 37958258 PMC10650064

[51] McDonald TJW Ratchford EV Henry-Barron BJ Kossoff EH Cervenka MC. Impact of the modified Atkins diet on cardiovascular health in adults with epilepsy. Epilepsy Behav. (2018) 79:82–6. doi: 10.1016/j.yebeh.2017.10.035, PMID: 29253679

[52] Jenkins DJ Wong JM Kendall CW Esfahani A Ng VW Leong TC. Effect of a 6-month vegan low-carbohydrate ('Eco-Atkins') diet on cardiovascular risk factors and body weight in Hyperlipidaemic adults: a randomised controlled trial. BMJ Open. (2014) 4:e003505. doi: 10.1136/bmjopen-2013-003505, PMID: 24500611 PMC3918974

