# Supplementary material for: Low-carbohydrate diet score and chronic obstructive pulmonary disease: a machine learning analysis of NHANES data
Source: Front Nutr. 2024 Dec 18;11:1519782. doi: 10.3389/fnut.2024.1519782 (PMC11706202; doi:10.3389/fnut.2024.1519782)

Supplementary Material

# Supplementary Tables

## Supplementary Table 1. Criteria for Calculating the LCD Score

| **Points** | **Carbohydrate/ total energy**  **%** | **Protein/ total energy**  **%** | **Fat/ total energy**  **%** |
| --- | --- | --- | --- |
| 0 | > 56.0 | <14.1 | <26.0 |
| 1 | 51.6-56.0 | 14.1-15.6 | 26.0-29.5 |
| 2 | 49.1-51.5 | 15.7-16.6 | 29.6-31.6 |
| 3 | 47.1-49.0 | 16.7-17.3 | 31.7-33.2 |
| 4 | 45.2-47.0 | 17.4-18.0 | 33.3-34.7 |
| 5 | 43.3-45.1 | 18.1-18.7 | 34.8-36.1 |
| 6 | 41.2-43.2 | 18.8-19.4 | 36.2-37.7 |
| 7 | 38.8-41.1 | 19.5-20.3 | 37.8-39.5 |
| 8 | 35.4-38.7 | 20.4-21.5 | 39.6-42.0 |
| 9 | 29.3-35 .3 | 21.6-24.0 | 42.1-46.9 |
| 10 | <29.3 | >24.0 | >46.9 |

The LCD score is the sum of the scores for three nutrients, with a total range from 0 to 30.

## Supplementary Table 2. Weighted Baseline Characteristics of Study Participants Stratified by LCD Score, Post-PSM

| **Characteristic** | **Overall**  (n = 3,764) | **Q1**  (n = 1,059) | **Q2**  (n = 945) | **Q3**  (n = 861) | **Q4**  (n = 899) | ***P*-value** |
| --- | --- | --- | --- | --- | --- | --- |
| COPD (%) | 1,882 (50%) | 558 (56%) | 494 (50%) | 414 (49%) | 416 (44%) | 0.001 |
| Age (year) | 60.23± (10.85) | 60.11± (10.98) | 60.25± (10.94) | 60.65± (10.71) | 59.96± (10.74) | 0.718 |
| Sex (%) |  |  |  |  |  | 0.059 |
| Male | 2,104 (53%) | 549 (49%) | 524 (53%) | 486 (53%) | 545 (58%) |  |
| Female | 1,660 (47%) | 510 (51%) | 421 (47%) | 375 (47%) | 354 (42%) |  |
| Race (%) |  |  |  |  |  | 0.328 |
| Mexican American | 220 (2.1%) | 70 (2.7%) | 54 (1.9%) | 48 (1.9%) | 48 (1.9%) |  |
| Other Hispanic | 211 (2.0%) | 70 (2.5%) | 57 (2.1%) | 50 (2.2%) | 34 (1.2%) |  |
| Non-Hispanic White | 2,355 (84%) | 631 (82%) | 604 (84%) | 525 (83%) | 595 (85%) |  |
| Non-Hispanic Black | 690 (6.8%) | 195 (6.8%) | 167 (6.4%) | 179 (7.7%) | 149 (6.4%) |  |
| Other Race | 288 (5.5%) | 93 (6.4%) | 63 (5.3%) | 59 (5.2%) | 73 (5.1%) |  |
| Education level (%) |  |  |  |  |  | 0.562 |
| <High school | 894 (16%) | 267 (18%) | 218 (15%) | 204 (16%) | 205 (15%) |  |
| High school | 1,051 (28%) | 292 (28%) | 271 (28%) | 242 (29%) | 246 (25%) |  |
| >High school | 1,819 (56%) | 500 (54%) | 456 (57%) | 415 (55%) | 448 (60%) |  |
| Marital status (%) |  |  |  |  |  | 0.724 |
| Unmarried | 3,329 (90%) | 937 (90%) | 843 (91%) | 763 (89%) | 786 (90%) |  |
| Married | 435 (10%) | 122 (10%) | 102 (8.9%) | 98 (11%) | 113 (10%) |  |
| PIR | 3.08± (1.63) | 2.88± (1.62) | 3.02± (1.65) | 3.10± (1.59) | 3.33± (1.62) | 0.002 |
| BMI (kg/m^2^) | 28.55± (6.13) | 27.81± (6.32) | 28.19± (5.80) | 28.72± (6.13) | 29.56± (6.12) | <0.001 |
| Waist circumference (cm) | 101.42± (15.48) | 99.32± (15.75) | 100.32± (14.93) | 101.65± (15.30) | 104.59± (15.40) | <0.001 |
| WHtR | 0.60± (0.09) | 0.59± (0.09) | 0.59± (0.09) | 0.60± (0.09) | 0.61± (0.09) | <0.001 |
| Physical activity (%) |  |  |  |  |  | 0.028 |
| Inactive | 2,959 (78%) | 834 (79%) | 733 (74%) | 683 (81%) | 709 (79%) |  |
| Active | 805 (22%) | 225 (21%) | 212 (26%) | 178 (19%) | 190 (21%) |  |
| Smoking status (%) |  |  |  |  |  | 0.304 |
| No | 1,008 (27%) | 285 (26%) | 274 (30%) | 226 (28%) | 223 (26%) |  |
| Yes | 2,756 (73%) | 774 (74%) | 671 (70%) | 635 (72%) | 676 (74%) |  |
| Hypertension (%) |  |  |  |  |  | 0.064 |
| No | 1,769 (52%) | 511 (54%) | 467 (53%) | 409 (54%) | 382 (47%) |  |
| Yes | 1,995 (48%) | 548 (46%) | 478 (47%) | 452 (46%) | 517 (53%) |  |
| Diabetes (%) |  |  |  |  |  | <0.001 |
| No | 2,952 (83%) | 874 (87%) | 751 (84%) | 682 (83%) | 645 (79%) |  |
| Yes | 812 (17%) | 185 (13%) | 194 (16%) | 179 (17%) | 254 (21%) |  |
| Congestive heart failure (%) |  |  |  |  |  | 0.354 |
| No | 3,461 (94%) | 979 (94%) | 862 (94%) | 801 (95%) | 819 (93%) |  |
| Yes | 303 (5.8%) | 80 (5.6%) | 83 (6.0%) | 60 (4.7%) | 80 (7.0%) |  |
| Coronary heart disease (%) |  |  |  |  |  | 0.758 |
| No | 3,376 (91%) | 957 (92%) | 849 (91%) | 773 (91%) | 797 (90%) |  |
| Yes | 388 (8.8%) | 102 (8.2%) | 96 (8.6%) | 88 (8.7%) | 102 (9.8%) |  |
| Heart disease (%) |  |  |  |  |  | 0.203 |
| No | 3,399 (92%) | 955 (91%) | 864 (94%) | 774 (93%) | 806 (91%) |  |
| Yes | 365 (7.6%) | 104 (8.8%) | 81 (6.0%) | 87 (6.8%) | 93 (8.7%) |  |
| Stroke |  |  |  |  |  | 0.467 |
| No | 3,497 (94%) | 982 (93%) | 879 (95%) | 801 (95%) | 835 (94%) |  |
| Yes | 267 (5.7%) | 77 (6.6%) | 66 (5.5%) | 60 (4.9%) | 64 (5.5%) |  |
| Magnesium intake (mg) | 298.41± (128.27) | 292.00± (125.41) | 298.53± (131.55) | 294.89± (117.84) | 308.52± (136.76) | 0.228 |
| Calcium intake (mg) | 934.73± (514.88) | 918.58± (484.53) | 943.17± (557.45) | 929.11± (504.51) | 948.99± (512.02) | 0.848 |
| Vitamin D intake (mcg) | 4.93± (5.26) | 4.45± (3.95) | 4.80± (4.79) | 5.07± (5.38) | 5.43± (6.62) | 0.189 |
| Fat intake score | 4.96± (3.91) | 1.10± (1.49) | 4.33± (3.42) | 6.53± (3.44) | 8.25± (2.61) | <0.001 |
| Protein intake score | 2.55± (3.10) | 0.72± (1.16) | 1.81± (2.50) | 2.68± (3.04) | 5.14± (3.38) | <0.001 |
| Carbohydrate intake score | 4.02± (3.16) | 1.08± (1.39) | 3.02± (2.74) | 4.71± (2.13) | 7.56± (1.76) | <0.001 |

Mean ± SD for continuous variables: P values were calculated using weighted analysis of variance (ANOVA). For categorical variables, n (%), P values were calculated using weighted Chi-square tests. Abbreviations: PIR, poverty-to-income ratio; BMI, body mass index; WHtR, waist-to-height ratio; LCD score, low-carbohydrate-diet score; COPD, chronic obstructive pulmonary disease.

# Supplementary Figures

## Supplementary Figure 1. Love plot of standardized mean differences before and after propensity score matching. This plot illustrates the standardized mean differences (SMD) for all baseline variables before and after propensity score matching. The reduction in SMD values post-matching, with all values close to or below 0.1, indicates successful balance between groups.


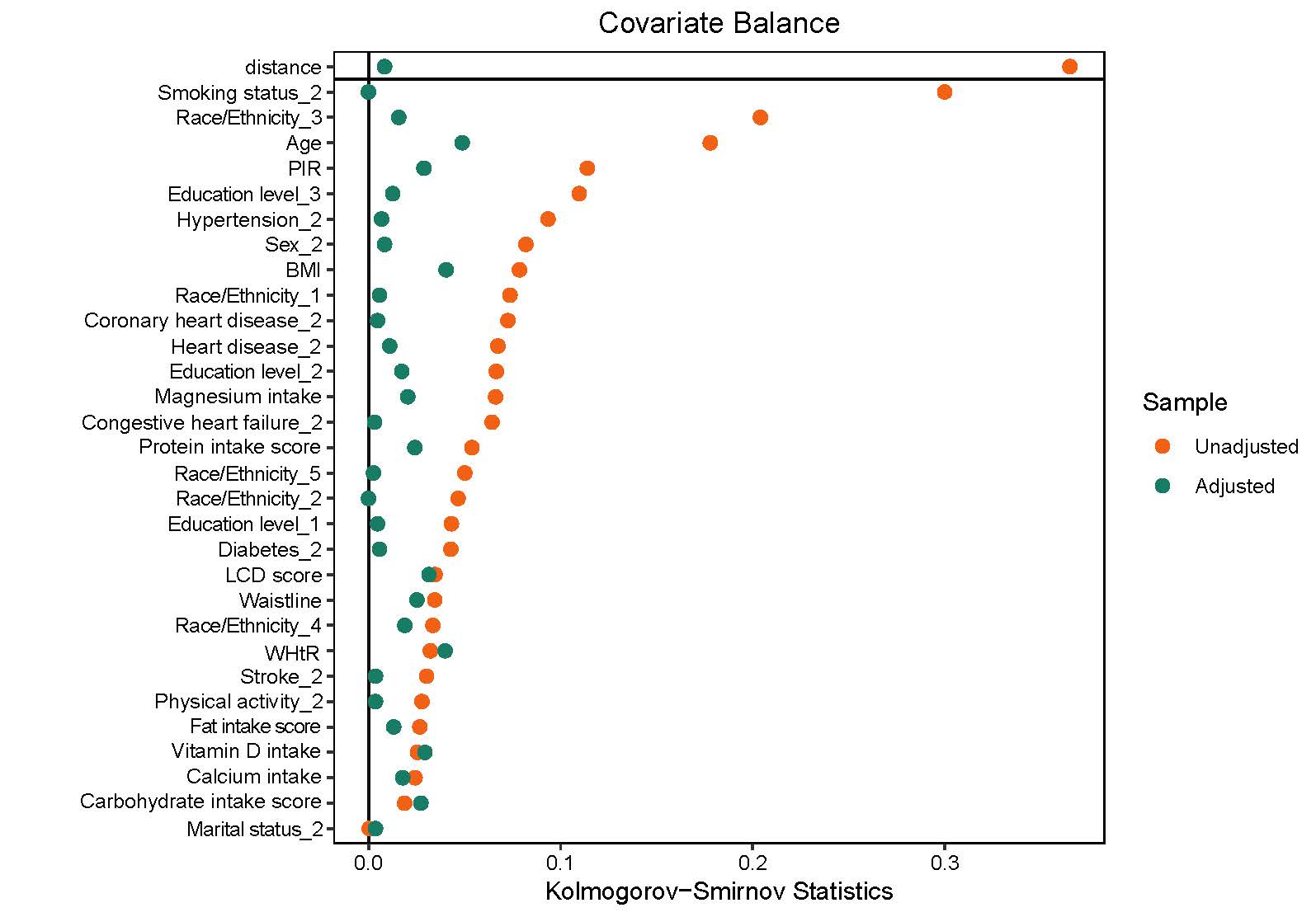


## Supplementary Figure 2. Histograms and density plots of baseline variables after propensity score matching. These visualizations depict the distributions of baseline variables between groups after propensity score matching. Matching variables included age, sex, race, education level, PIR, BMI, WHtR, smoking status, hypertension, diabetes, congestive heart failure, coronary heart disease, heart disease, stroke, and magnesium intake. The overlapping distributions confirm improved balance and comparability of the variables post-matching.


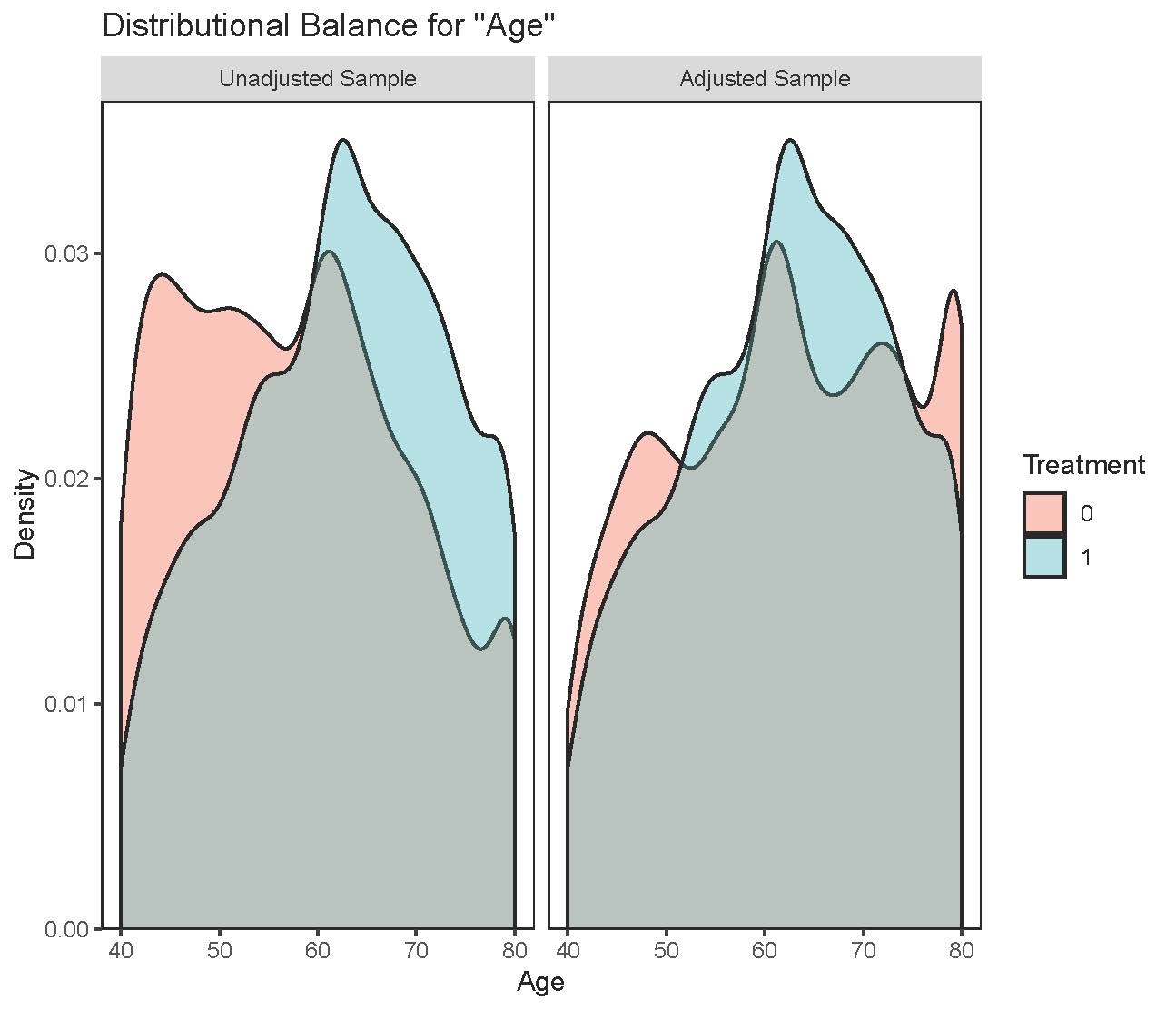

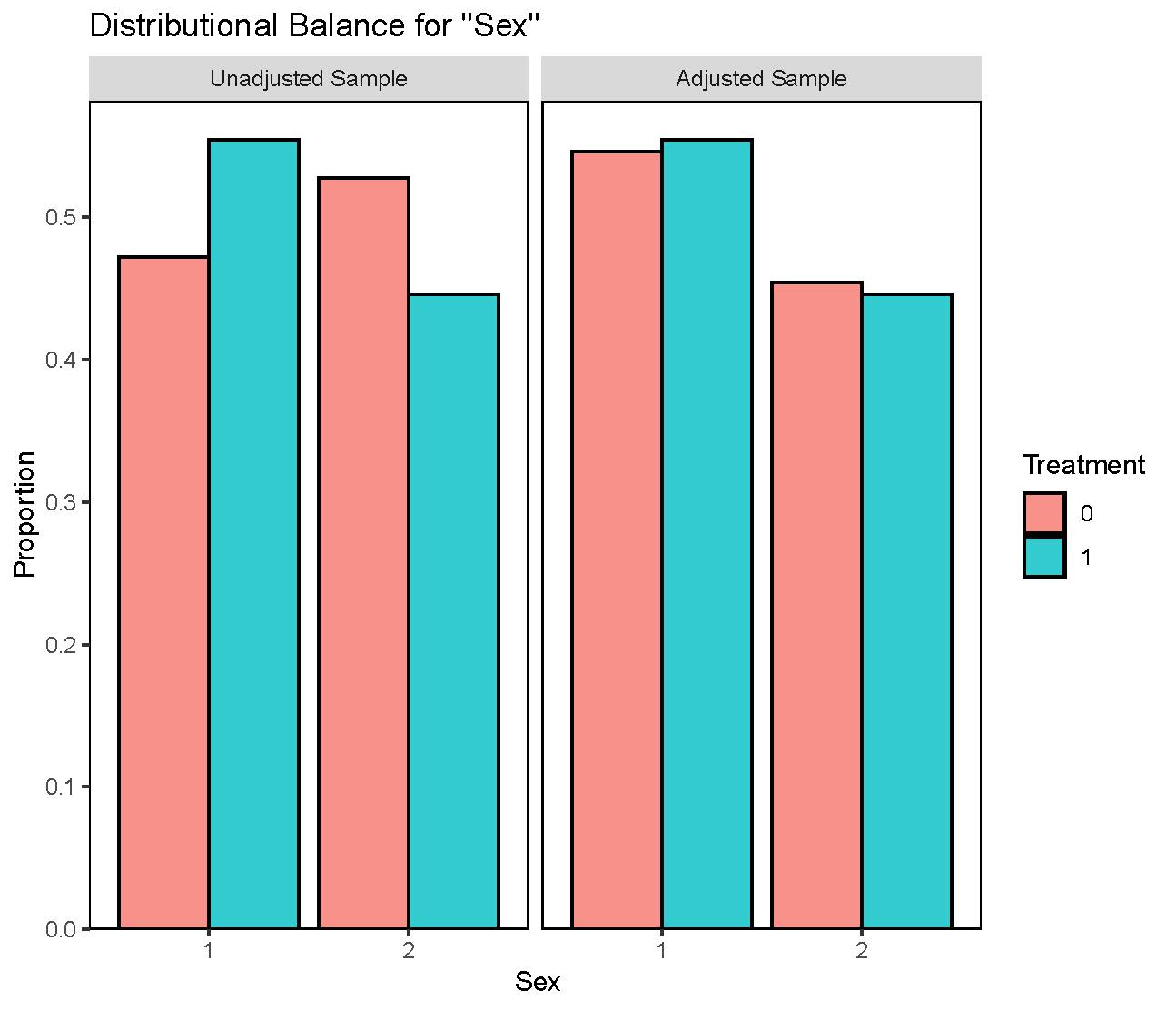

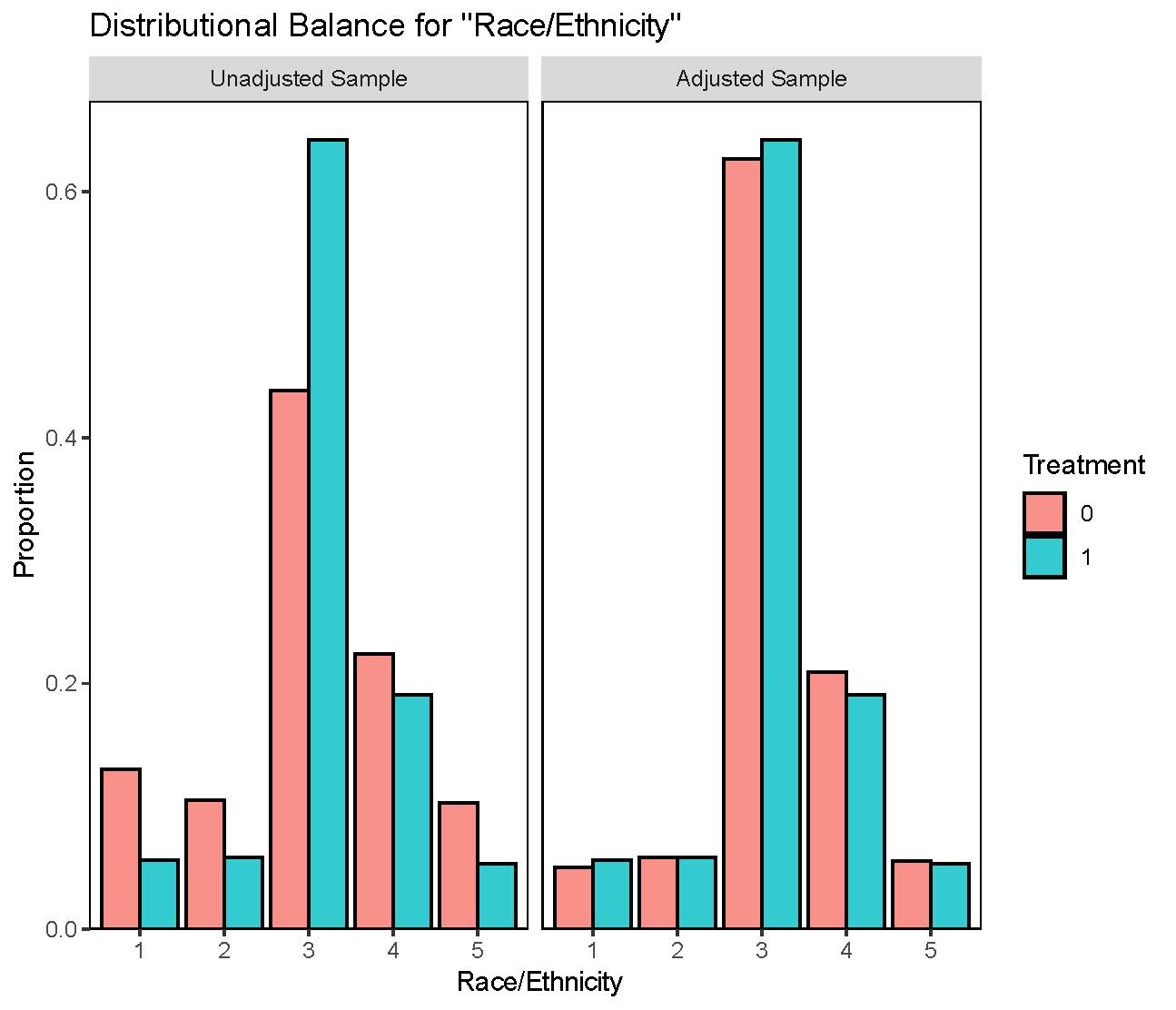

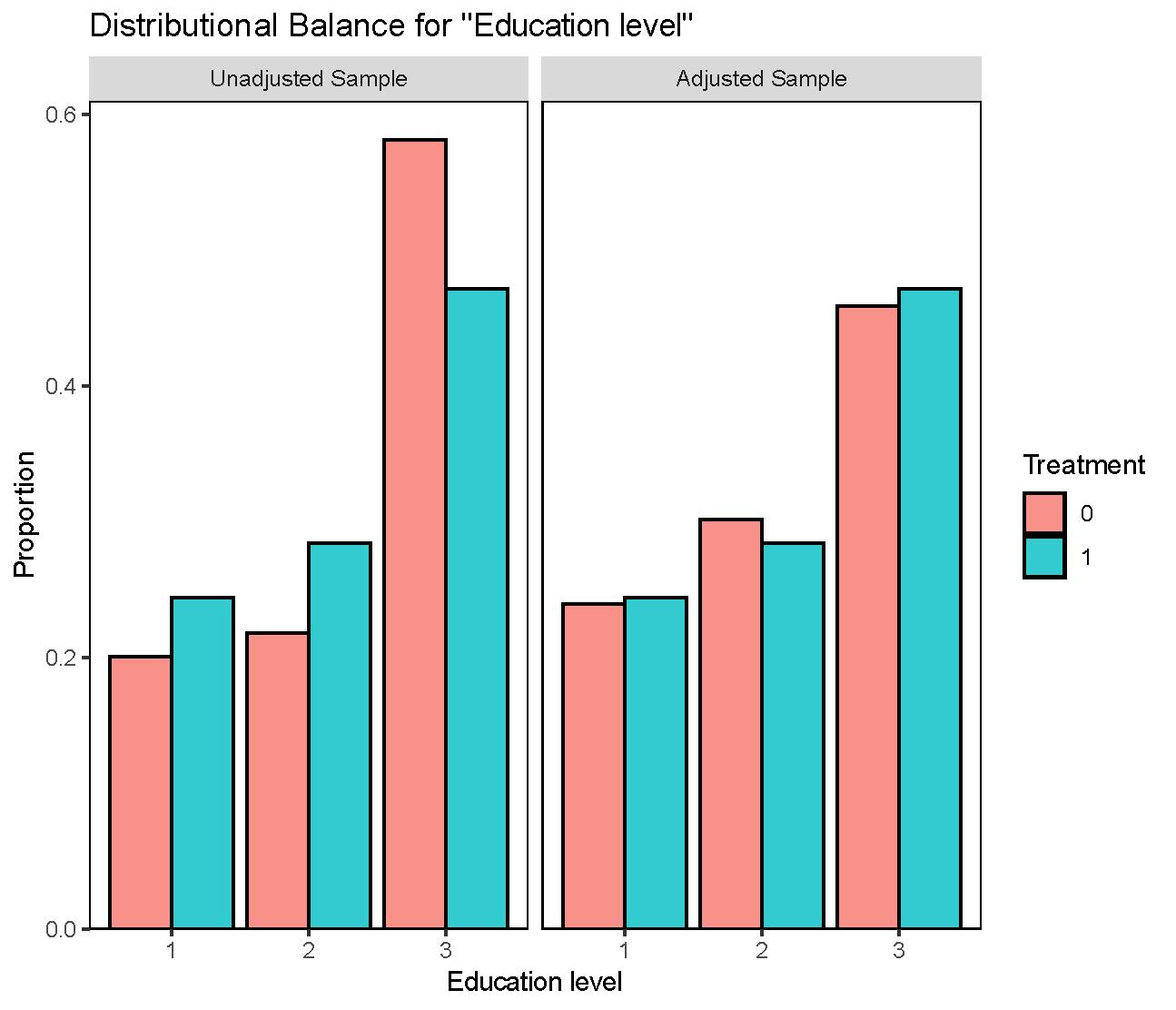

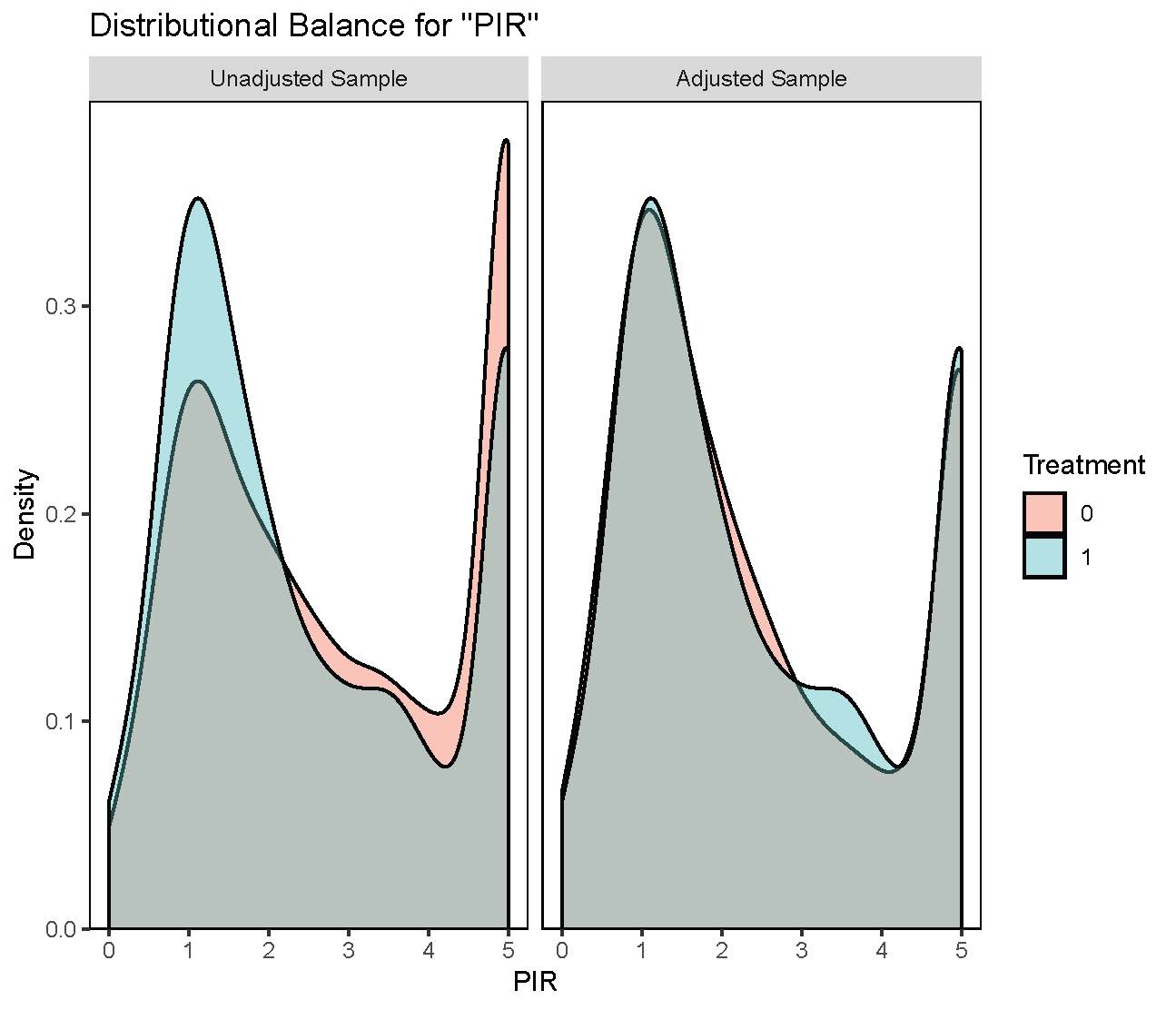

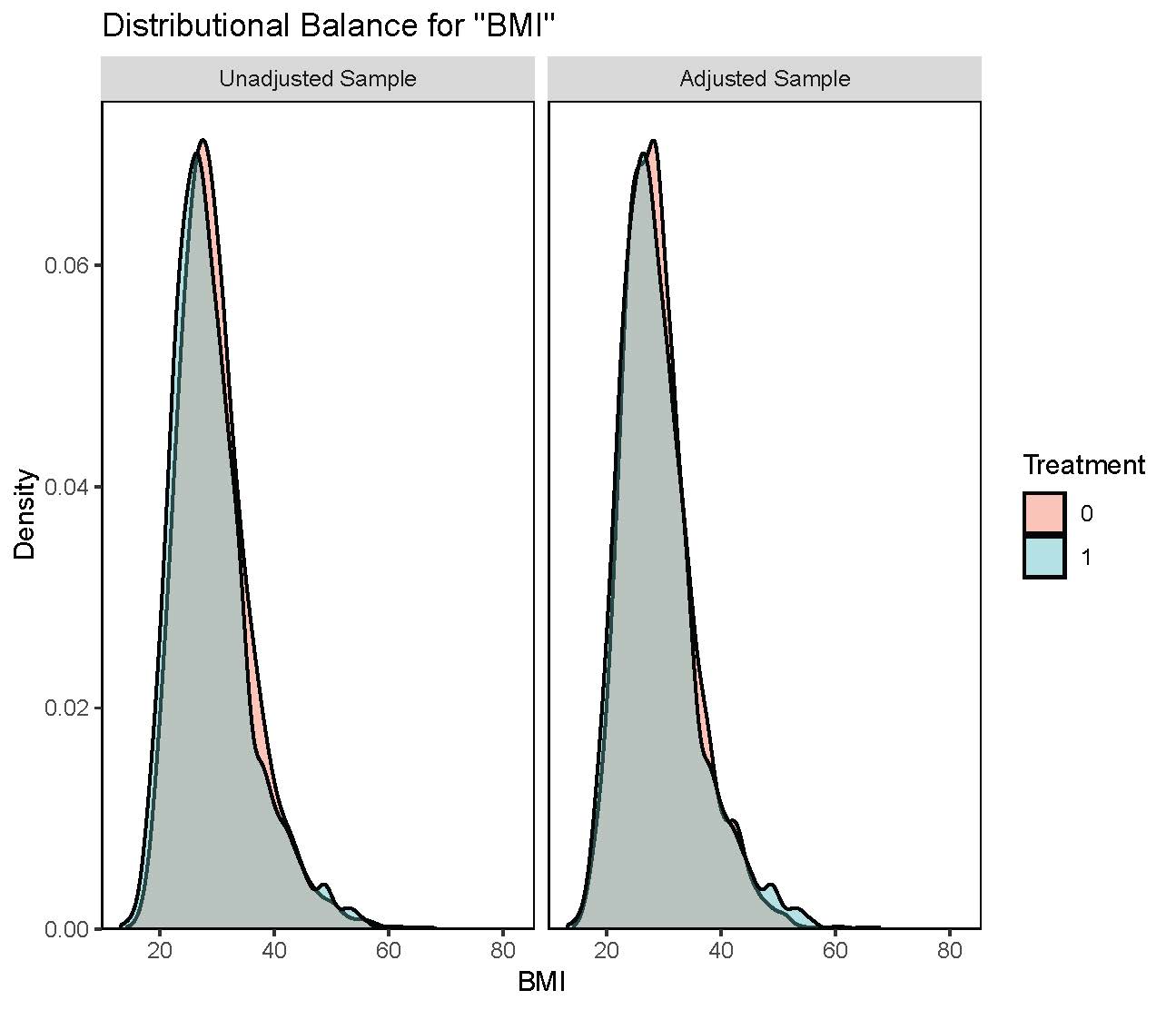

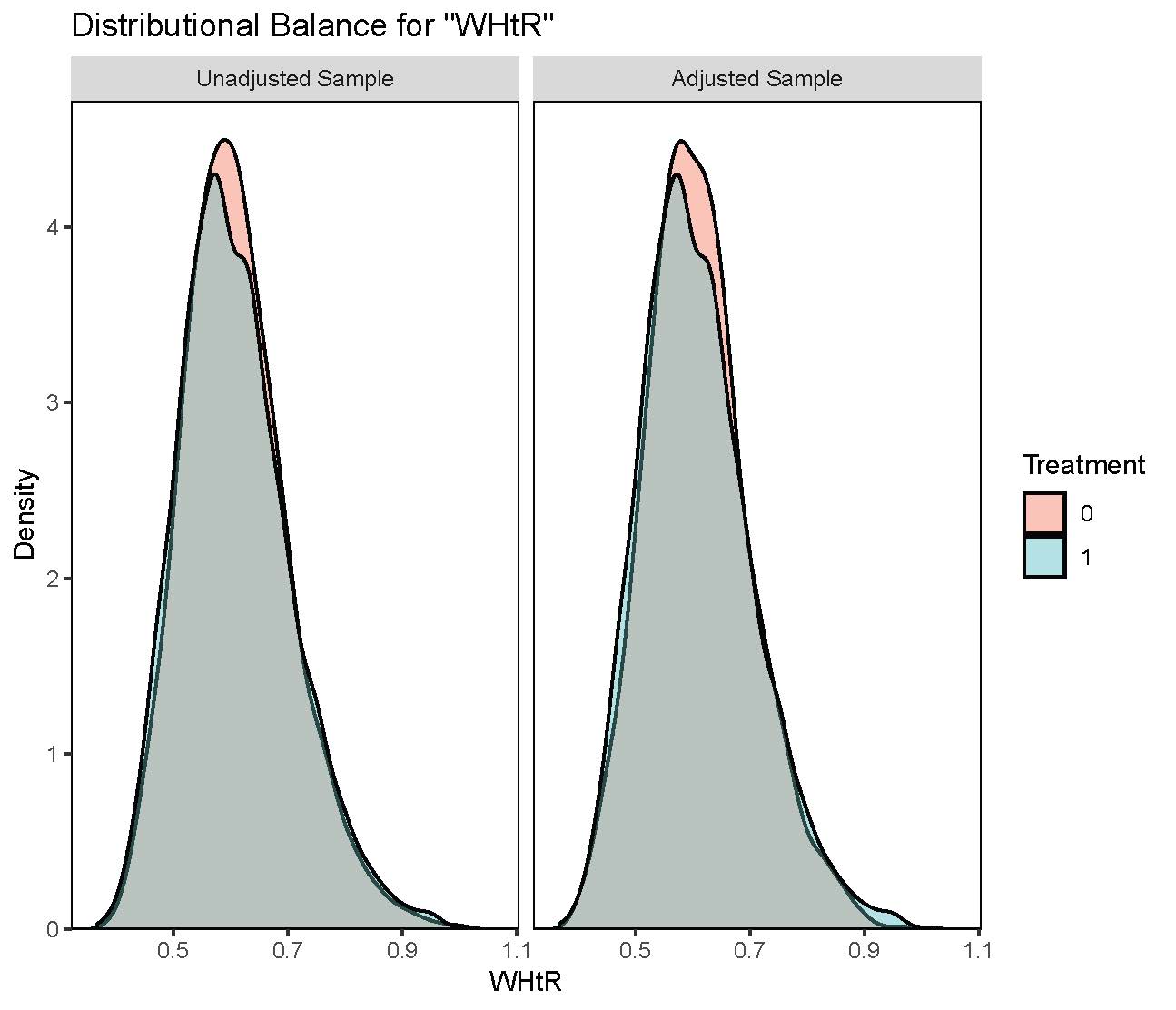

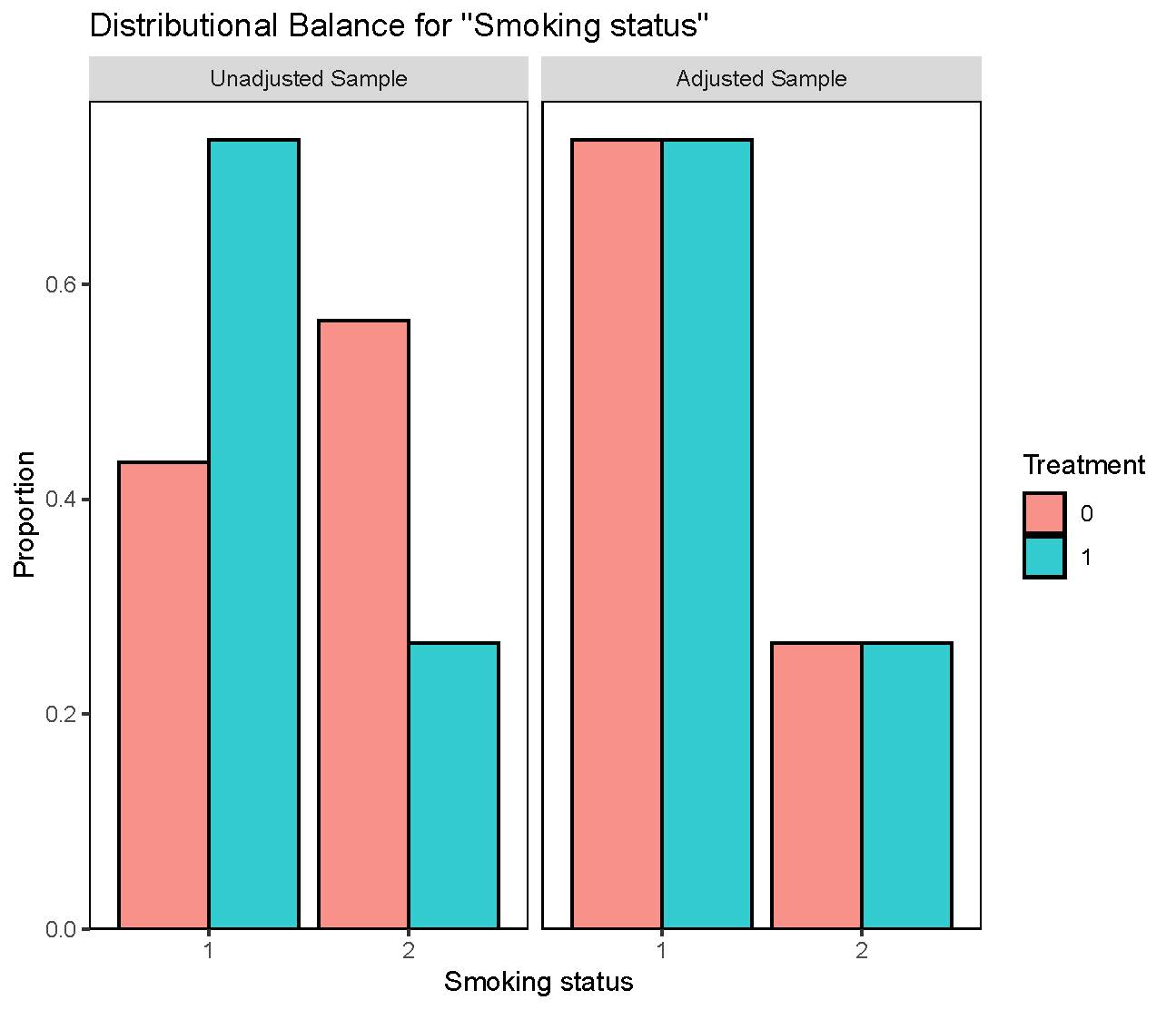

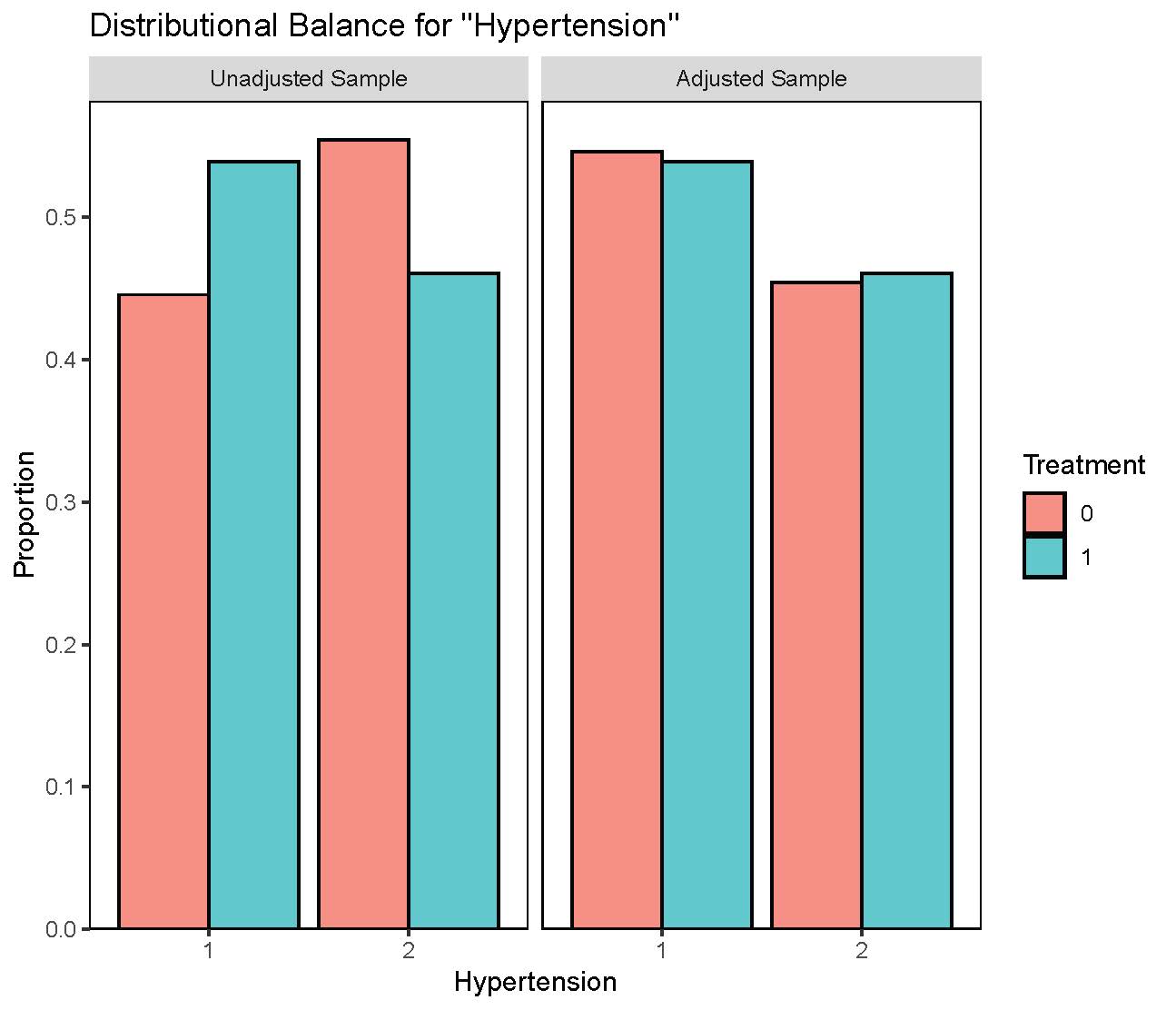

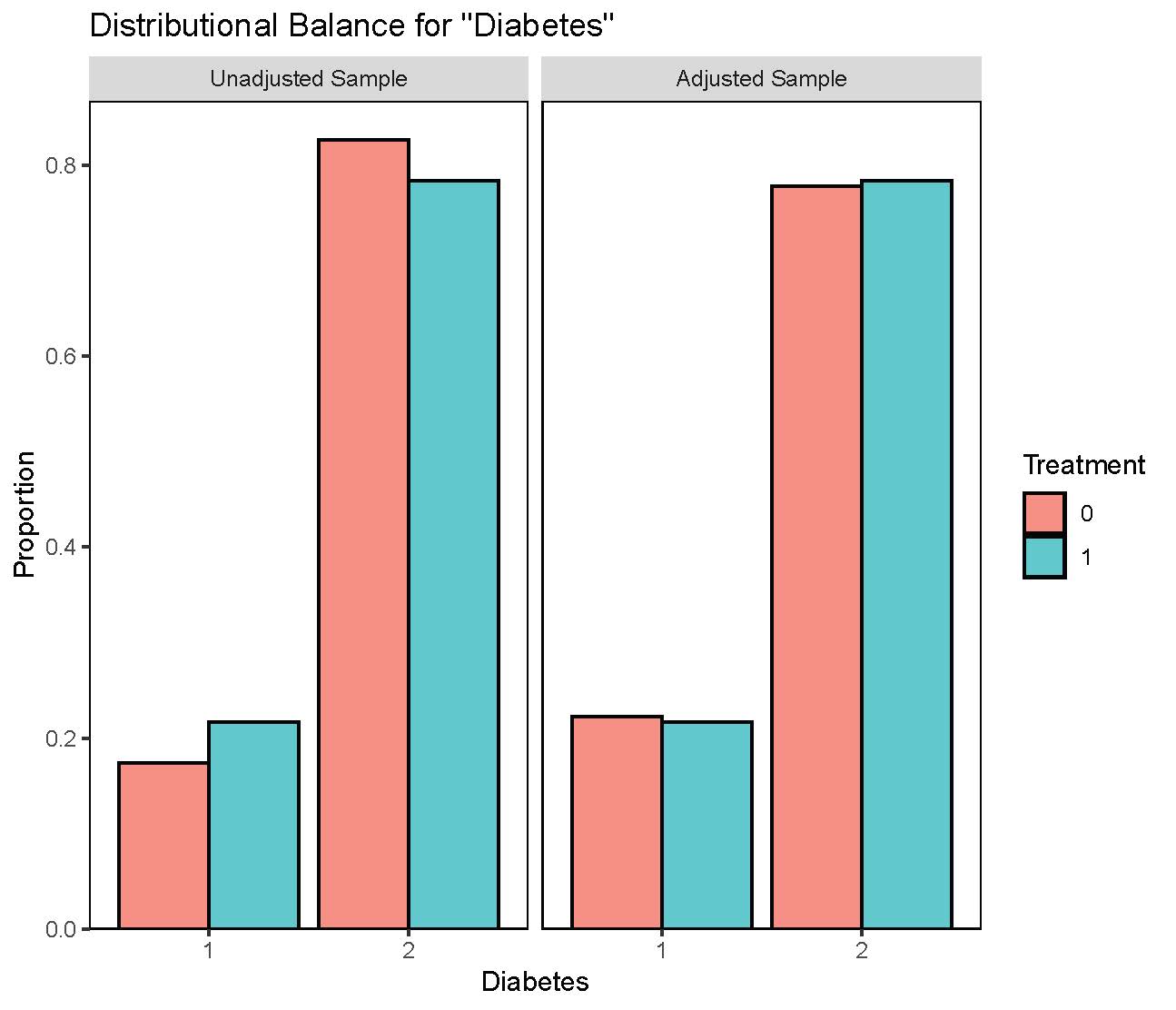

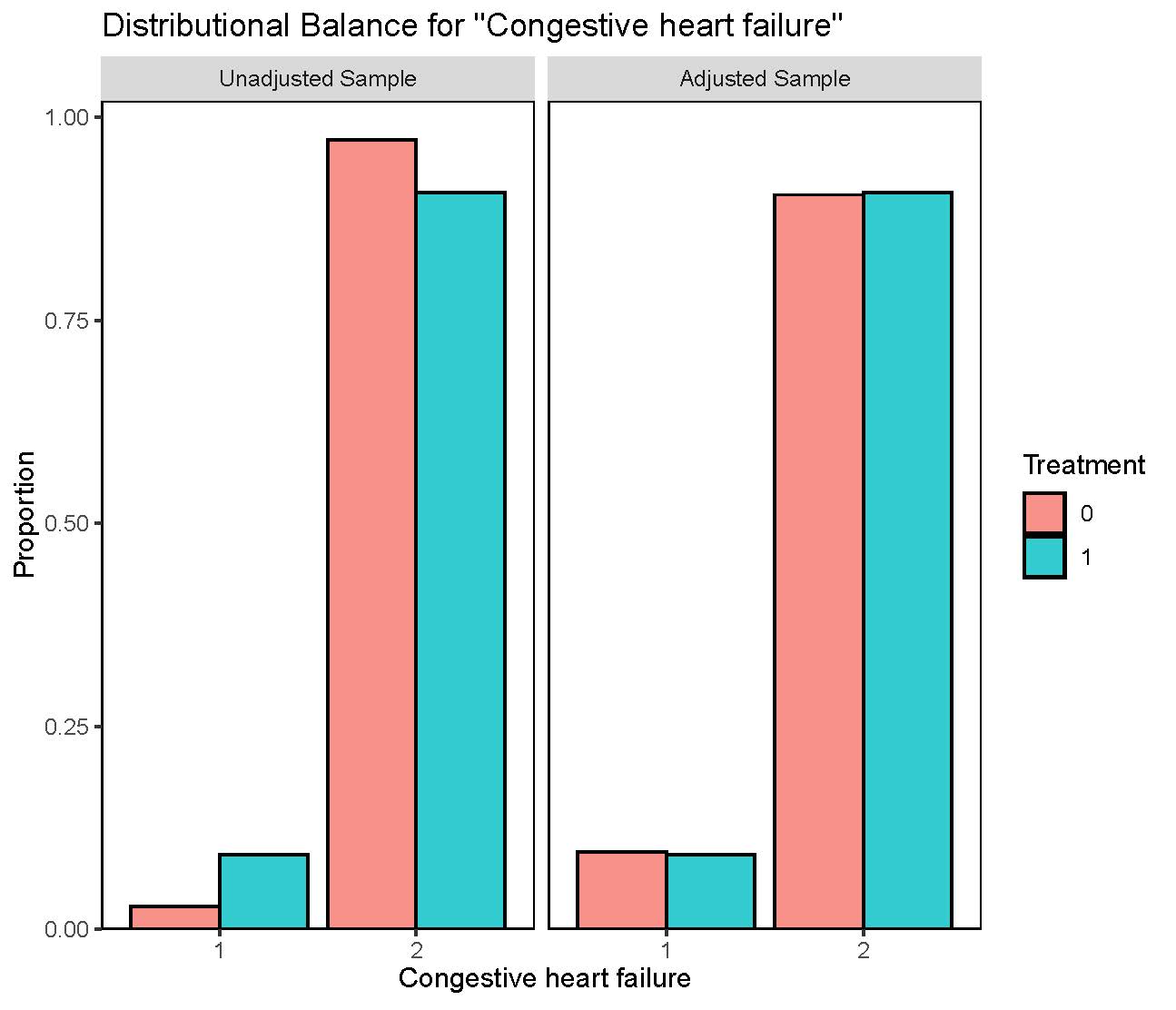

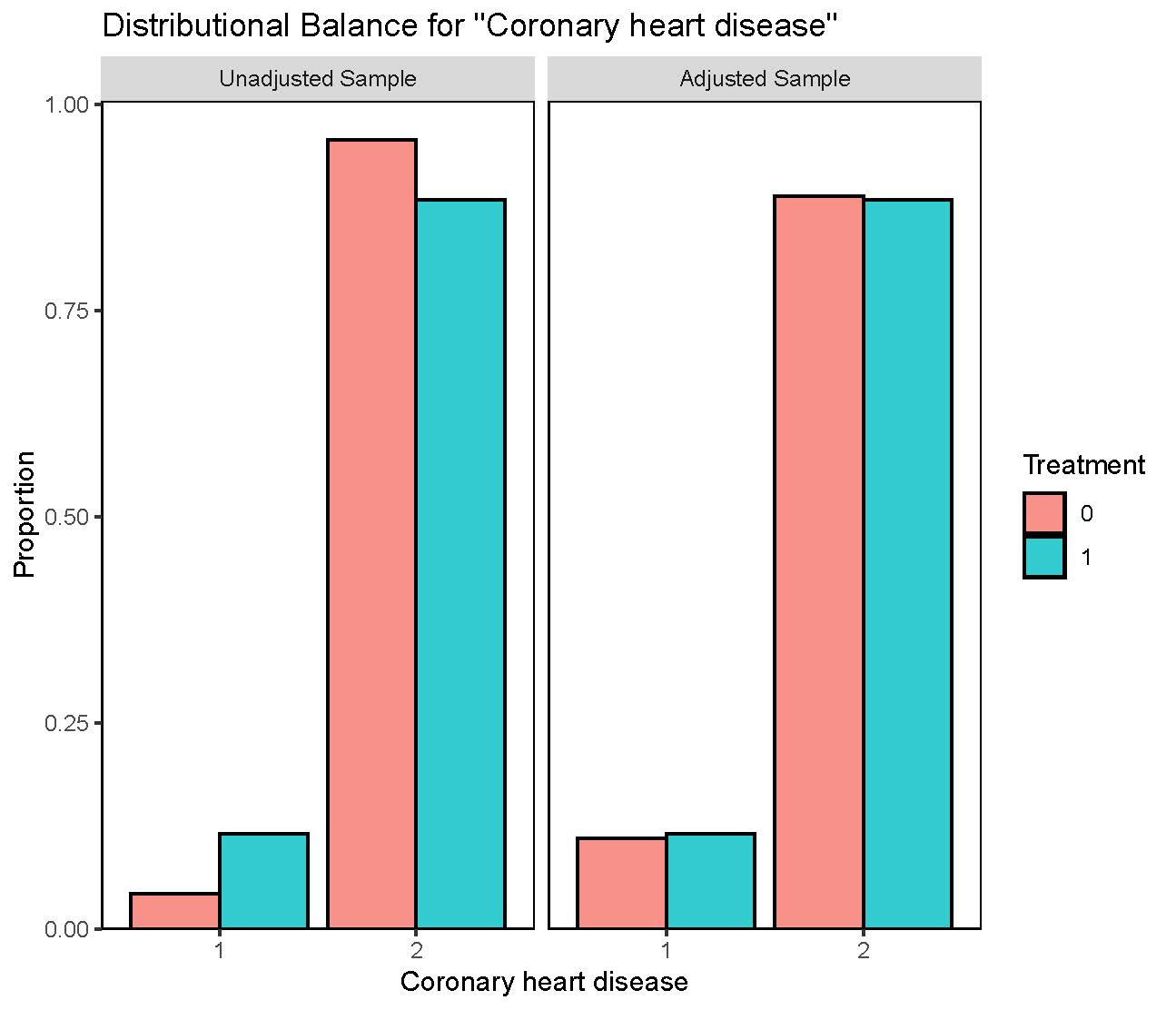

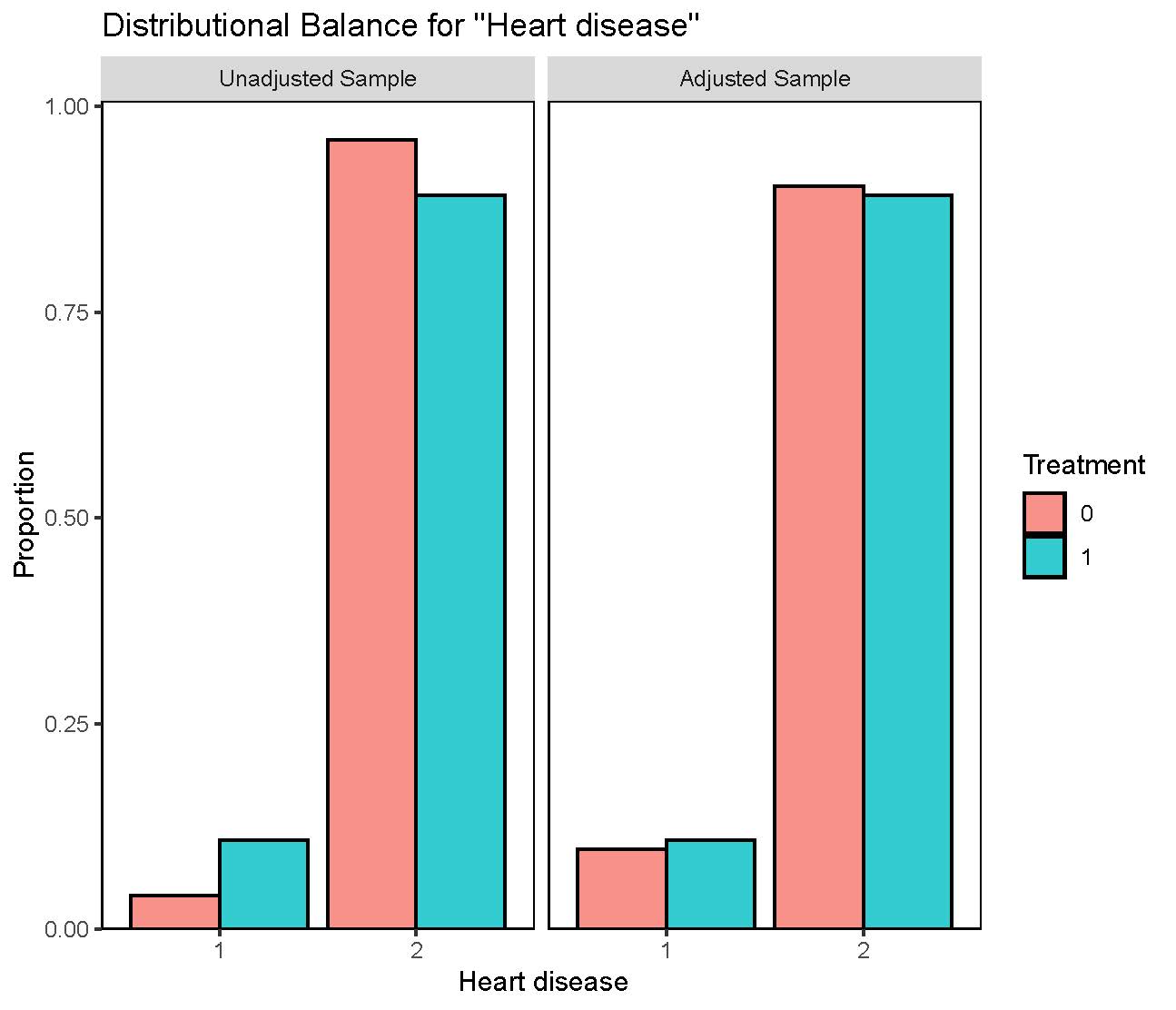

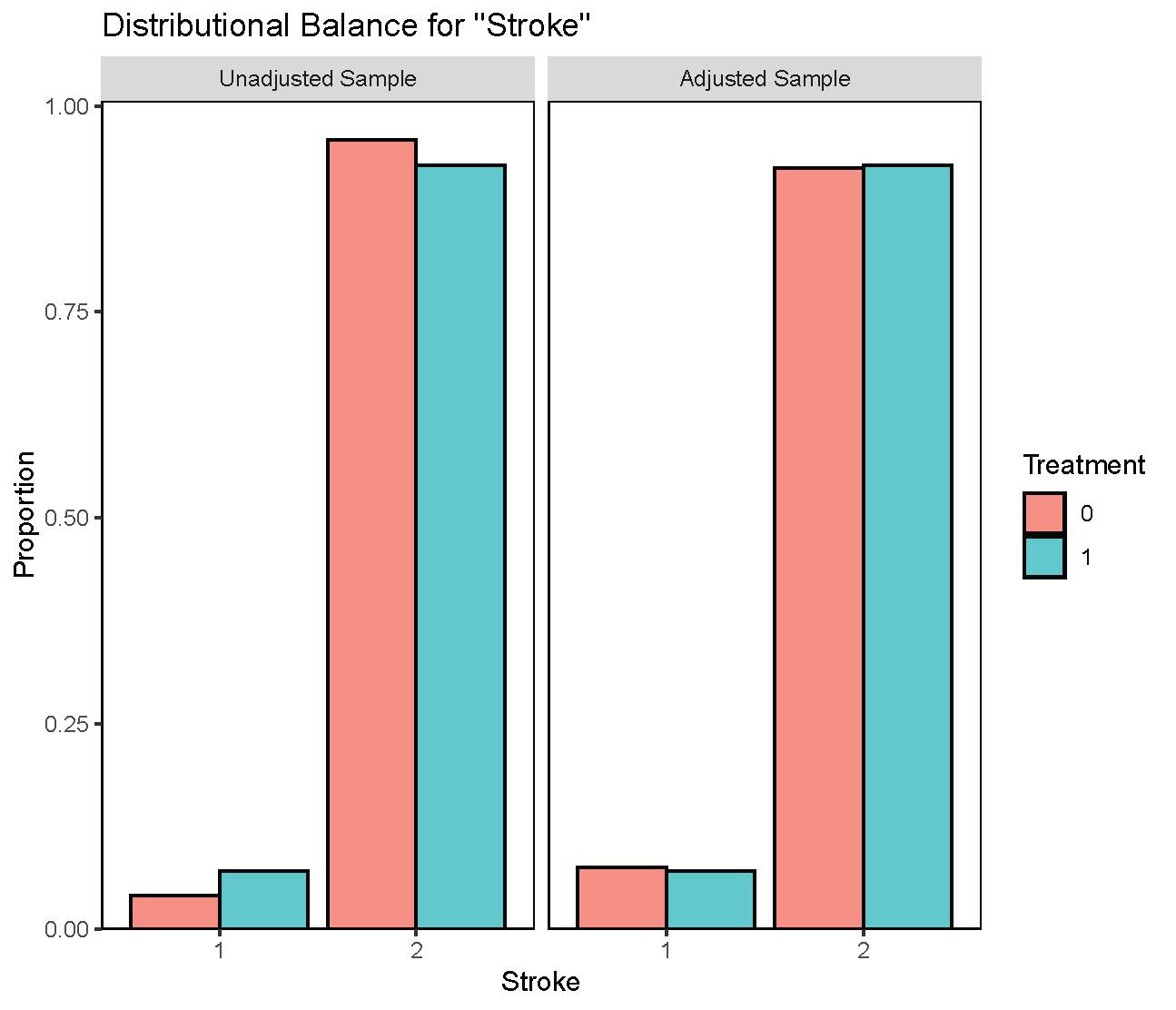

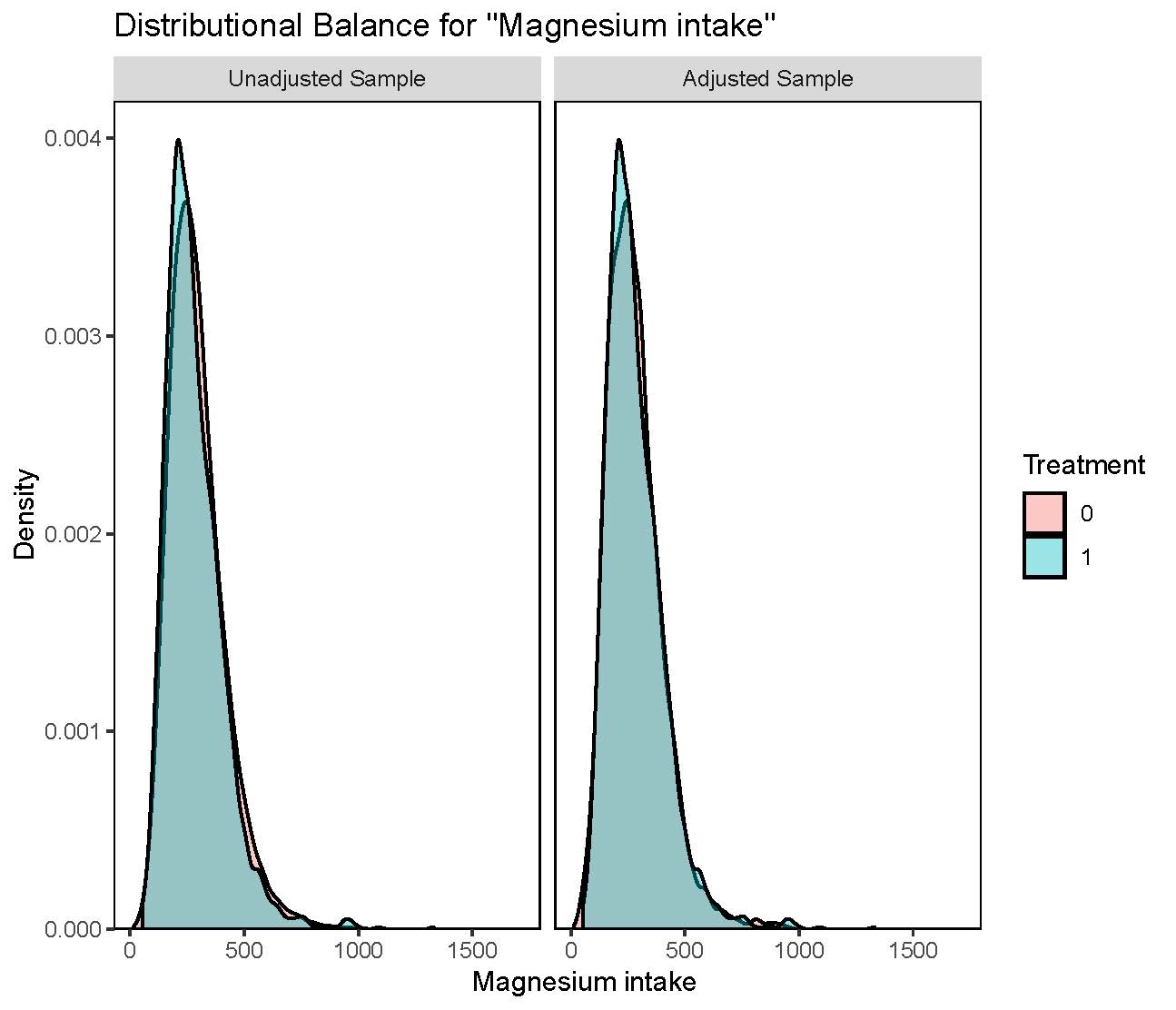

Supplement: Supplementary file 2 [file Data_Sheet_2.docx]
